# Supplementary material for: Prognostic value of acute National Institutes of Health Stroke Scale Items on disability: a registry study of first-ever stroke in the western part of Sweden
Source: BMJ Open. 2023 Dec 18;13(12):e080007. doi: 10.1136/bmjopen-2023-080007 (PMC10748889; doi:10.1136/bmjopen-2023-080007)
Supplement: Supplementary data [file bmjopen-2023-080007supp001.pdf]

Supplemetary table 1. Logistic regression; dichotomized NIHSS items sub-score stratified for stroke subtypes, and outcome mRS≤1

	Ischemic stroke				Hemorrhage			
	Crude		Adjusted§		Crude		Adjusted†	
NIHSS	OR	95% CI	OR	95% CI	OR	95% CI	OR	95% CI
1a Consciousness	0.29	0.17-0.51	0.70	0.37-1.30	0.21	0.05-0.97	0.83	0.13-5.30
1b Orientation (0 ref.)	0.34	0.25-0.45	0.83	0.58-1.21	0.52	0.23-1.18	1.42	0.48-4.18
1c Commands	0.31	0.19-0.50	0.90	0.49-1.66	0.21	0.05-0.99	0.31	0.03-2.76
2 Best Gaze	0.29	0.19-0.42	*0.57*	*0.34-0.94*	0.77	0.29-2.06	2.36	0.59-9.41
3 Visual field	*0.34*	*0.25-0.47*	*0.54*	*0.36-0.82*	0.97	0.44-2.16	1.54	0.55-4.30
4 Facial Palsy	0.55	0.43-0.69	0.89	0.66-1.22	0.59	0.27-1.29	1.81	0.59-5.57
5 Motor Arm								
Right	0.33	0.21-050	0.60	0.35-1.02	*0.32*	*0.11-0.90*	0.56	0.14-2.17
Left	0.38	0.27-0.53	0.73	0.48-1.11	0.53	0.22-1.31	1.19	0.37-3.85
6 Motor Leg								
Right	*0.27*	*0.18-0.41*	*0.58*	*0.35-0.97*	0.37	0.13-1.08	0.63	0.16-2.45
Left	0.38	0.27-0.54	0.69	0.45-1.05	0.50	0.21-1.17	0.95	0.30-3.08
7 Ataxia	0.49	0.36-0.68	0.71	0.49-1.03	0.94	0.35-2.59	1.01	0.30-3.38
8 Sensory	0.55	0.42-0.72	0.85	0.61-1.20	0.28	0.12-061	0.42	0.16-1.12
9 Best Language	0.43	0.33-0.57	0.95	0.66-1.36	0.30	0.13-0.70	0.58	0.21-1.60
10 Dysarthria	0.50	0.39-0.63	0.86	0.63-1.18	0.29	0.13-064	0.46	0.17-1.24
11 Neglect	0.36	0.25-0.52	0.75	0.47-1.19	0.69	0.29-1.64	1.83	0.57-5.83
Motor arm symptoms	0.31	0.24-0.42	*0.56*	*0.37-0.84*	0.30	0.14-0.64	0.60	0.19-1.91
Motor leg symptoms	0.30	0.23-0.40	*0.53*	*0.36-0.79*	0.33	0.15-0.72	0.60	0.18-1.94
Right motor symptoms	0.30	0.21-0.44	0.63	0.40-1.00	0.39	0.16-0.99	0.74	0.23-2.38
Left motor symptoms	0.40	0.29-0.54	0.69	0.48-1.00	0.51	0.22-1.16	0.90	0.29-2.76
Motor symptoms in at least one limb	0.32	0.25-0.41	0.55	0.38-0.79	0.32	0.15-0.68	0.63	0.20-2.05

§Adjusted for age, gender, premorbid independency, stroke severity and reperfusion therapy
† Adjusted for age, gender, premorbid independency and stroke severity
NIHSS=National Institutes of Health Stroke Scale, OR=odds ratio, CI=confidence interval
Each NIHSS item component on admission was dichotomized into; no symptoms (score 0) or symptoms (score of 1 or more).
Significant values in Italic.

Supplemental table 2: Characteristics of patients not included

**Total**		**2269**
**Age**		
	**Mean (SD)**	75 (14)*
	**Median (IQR)**	77 (85-67)
**Sex (female) (%)**		1150 (51)*
**Stroke type (%)***		
	**Ischemic**	1955 (86)
	**Hemorrhagic**	350 (13)
**Reperfusion therapy (%)**		242 (16.5)
**NIHSS**		
	**Mean (SD)**	7 (8)*
	**Median (IQR)**	2 (8-0)
**Stroke severity (%)***		
	**Mild (NIHSS≤3)**	1107 (49)
	**Mild to moderate**	777 (34)
	**Severe (NIHSS>15)**	380 (17)
**mRS 3 months (n = 293)**		
	**Median (IQR)**	2 (3-1)
	**Excellent outcome (mRS≤1)**	43 (15)*
**Discharged to (%)**		
	**Home**	1252 (55)*
**Premorbid independency* (%)**		1169 (79.5)
**NIHSS sub score (%)**		
	**1a Consciousness (n = 1382)**	125 (9)*
	**1b Orientation (n = 1366)**	410 (30)*
	**1c Commands (n = 1357)**	170 (13)*
	**2 Best Gaze (n = 1329)**	240 (18)*
	**3 Visual field (n = 1304)**	280 (21)*
	**4 Facial palsy (n = 1332)**	454 (30.9)*
	**5 Motor arm**	
	**Right (n = 1354)**	213 (16)*
	**Left (n = 1356)**	220 (16)
	**6 Motor leg**	
	**Right (n = 1348)**	222 (16)*
	**Left (n = 1350)**	229 (17)*
	**7 Ataxia (n = 1313)**	232 (18)
	**8 Sensory (n = 1321)**	386 (29)*
	**9 Best Language (n = 1317)**	422 (32)*
	**10 Dysarthria (n = 1310)**	464 (35)*
	**11 Neglect (n = 1256)**	194 (15)
**Cardiovascular risk/comorbidity (%)**		
	**Atrial fibrillation**	450 (20)*
	**Diabetes**	408 (18)
	**Smoking**	291 (13)*
	**On statins**	504 (22)
	**Hypertension**	1210 (53)*

*signficant different from included cases. SD standard deviation, IQR interquartile range, NIHSS=National Institutes of Health Stroke Scale, mRS=modified Rankin scale

Hagberg G, et al. BMJ Open 2024; 13:e080007. doi: 10.1136/bmjopen-2023-080007
