# Supplementary material for: Prognostic value of acute National Institutes of Health Stroke Scale Items on disability: a registry study of first-ever stroke in the western part of Sweden
Source: BMJ Open. 2023 Dec 18;13(12):e080007. doi: 10.1136/bmjopen-2023-080007 (PMC10748889; doi:10.1136/bmjopen-2023-080007)
Supplement: Author's manuscript [file bmjopen-2023-080007.draft_revisions.pdf]

BMJ Open

BMJ Open is committed to open peer review. As part of this commitment we make the peer review history of every article we publish publicly available.

When an article is published we post the peer reviewers' comments and the authors' responses online. We also post the versions of the paper that were used during peer review. These are the versions that the peer review comments apply to.

The versions of the paper that follow are the versions that were submitted during the peer review process. They are not the versions of record or the final published versions. They should not be cited or distributed as the published version of this manuscript.

BMJ Open is an open access journal and the full, final, typeset and author-corrected version of record of the manuscript is available on our site with no access controls, subscription charges or pay-per-view fees (http://bmjopen.bmj.com).

If you have any questions on BMJ Open's open peer review process please email info.bmjopen@bmj.com

The prognostic value of acute National Institutes of Health Stroke Scale Items on disability: A Registry Study of first-ever-stroke

Journal:	*BMJ Open*
Manuscript ID	bmjopen-2023-080007
Article Type:	Original research
Date Submitted by the Author:	18-Sep-2023
Complete List of Authors:	Hagberg, Guri; Oslo universitetssykehus Ullevål, Neurological Department ; University of Gothenburg Institute of Neuroscience and Physiology, Section for Clinical Neuroscience and Rehabilitation<br>Ihle-Hansen, Haakon; Vestre Viken HF, Department of medical research; University of Oslo, Institute of Clinical Medicine<br>Abzhandadze, Tamar; University of Gothenburg, Department of Clinical Neuroscience, Rehabilitation Medicine; Sahlgrenska University Hospital Department of Occupational Therapy and Physiotherapy<br>Reinholsson, Malin; University of Gothenburg Institute of Neuroscience and Physiology; Sahlgrenska University Hospital Department of Occupational Therapy and Physiotherapy<br>Hansen, Hege; Vestre Viken HF, Department of Medicine; Oslo University Hospital, Department of Neurology<br>Sunnerhagen, Katharina; University of Gothenburg Institute of Neuroscience and Physiology, Institute of Neuroscience and Physiology
Keywords:	Stroke < NEUROLOGY, Prognosis, Stroke medicine < INTERNAL MEDICINE, REGISTRIES

SCHOLARONE™
Manuscripts

BMJ

I, the Submitting Author has the right to grant and does grant on behalf of all authors of the Work (as defined in the below author licence), an exclusive licence and/or a non-exclusive licence for contributions from authors who are: i) UK Crown employees; ii) where BMJ has agreed a CC-BY licence shall apply, and/or iii) in accordance with the terms applicable for US Federal Government officers or employees acting as part of their official duties; on a worldwide, perpetual, irrevocable, royalty-free basis to BMJ Publishing Group Ltd ("BMJ") its licensees and where the relevant Journal is co-owned by BMJ to the co-owners of the Journal, to publish the Work in this journal and any other BMJ products and to exploit all rights, as set out in our licence.

The Submitting Author accepts and understands that any supply made under these terms is made by BMJ to the Submitting Author unless you are acting as an employee on behalf of your employer or a postgraduate student of an affiliated institution which is paying any applicable article publishing charge ("APC") for Open Access articles. Where the Submitting Author wishes to make the Work available on an Open Access basis (and intends to pay the relevant APC), the terms of reuse of such Open Access shall be governed by a Creative Commons licence – details of these licences and which Creative Commons licence will apply to this Work are set out in our licence referred to above.

Other than as permitted in any relevant BMJ Author's Self Archiving Policies, I confirm this Work has not been accepted for publication elsewhere, is not being considered for publication elsewhere and does not duplicate material already published. I confirm all authors consent to publication of this Work and authorise the granting of this licence.

For peer review only

The prognostic value of acute National Institutes of Health Stroke Scale Items on disability: A Registry Study of first-ever-stroke

MD PhD Guri Hagberg[1,3] MD PhD Håkon Ihle-Hansen[2], OT PhD Tamar Abzhandadze[3,4,5], PT MSc Malin Reinholdsson[3,4], MD PhD Hege Ihle-Hansen[1,2], Prof Katharina S. Sunnerhagen[3,6]

[1]Oslo Stroke Unit, Department of Neurology, Oslo University Hospital, Ullevål, Norway.

[2]Bærum Hospital- Vestre Viken Hospital Trust, Department of Medicine, Drammen, Norway.

[3]Institute of Neuroscience and Physiology, Section for Clinical Neuroscience and Rehabilitation, Gothenburg University, Gothenburg, Sweden

[4]Department of Occupational Therapy and Physiotherapy, Sahlgrenska University Hospital, Gothenburg, Sweden

[5]Division of Clinical Geriatrics, Department of Neurobiology, Care Sciences and Society, Karolinska Institutet, Stockholm, Sweden

[6]Neurocare, Sahlgrenska University Hospital, Gothenburg, Sweden.

Corresponding author: Guri Hagberg, Institute of Neuroscience and Physiology, Section for Clinical Neuroscience and Rehabilitation, Gothenburg University, Gothenburg, Sweden.

Mail: guhagb@ous-hf.no

Running title: The prognostic value of NIHSS sub-items

Keywords: Stroke, NIHSS, mRS, prognosis

Word count: 2475

For peer review only

Abstract

Objectives: The evidence of whether the individual items of the National Institutes of Health Stroke Scale (NIHSS) have the same weight for explaining the level of global disability after stroke is limited. We aimed to study how the individual items of NIHSS at admission relate to functional independence three months post-stroke in a comprehensive stroke register.

Materials and Methods: This cross-sectional and retrospective register-based study included patients with first-ever acute stroke admitted to three Swedish stroke units from November 2014 to August 2018, with available NIHSS at admission and modified Rankin Scale (mRS) at three months follow-up. The explanatory variables were NIHSS items and the outcome variable mRS. An excellent outcome was defined as mRS ≤1p.

Results: We included 1471 patients, mean age was 72 ± (14.5) years, 48 % were female, and 66 % had mild strokes (NIHSS ≤ 3). In adjusted binary logistic regression analysis, the NIHSS items impaired right motor arm and leg, and impairment in visual field, reduced the odds of an excellent outcome at three months (OR 0.60 (95% CI: 0.37-0.98), OR 0.60 (95% CI: 0.37-0.97), and OR 0.65 (95% CI: 0.45-0.94)).When exploring the strength of the relationship between NIHSS items and mRS ≤1p, orientation, language, and right leg motor had the highest, yet low correlation.

Conclusions: Stroke patients with scores on the NIHSS items right motor symptoms or visual field at admission are less likely to have excellent outcome at three months. Clinicians should consider the NIHSS items affected, not only the total NIHSS score, both in treatment guidance and prognostics.

Key words: Stroke, NIHSS, prognosis, mRS

Strengths and limitations of this study

- To our knowledge, we are the first to demonstrate the prognostic merit of NIHSS individual items regardless of stroke severity on post-stroke functioning.
- Using data from a stroke register of high quality and high coverage, reflecting real-life clinical setting, the generalizability of the findings are substantial.
- Since 40 % of the patients do not have mRS assessed at three months, there is still lack in knowledge of stroke outcome.

For peer review only - http://bmjopen.bmj.com/site/about/guidelines.xhtml

Introduction

The National Institutes of Health Stroke Scale (NIHSS) includes 15 individual items that measure motor and sensory function, vision, level of consciousness, attention, neglect, language, and speech production (1, 2). The NIHSS is the most widespread clinical scale used in patients presenting with acute stroke, and a high total NIHSS score is associated with poor outcomes in both hemorrhagic and ischemic strokes (3-5).

The total NIHSS score has several limitations as predictor of later stroke outcomes. The 15 items are weighted similarly, scoring 0-42, but left- brain strokes score in general 4 points higher on the NIHSS than right brain strokes of larger volume (6). Total NIHSS scores at admission are inferior to 24 h and discharge scores in predicting post-stroke functional outcomes, due to spontaneous improvement and effect of acute interventions like thrombolysis or thrombectomy (7, 8). In strokes affecting posterior circulation, a low NIHSS might not predict a good functional outcome as the NIHSS is biased towards anterior circulation strokes (9, 10). In addition, in minor stroke, defined as NIHSS ≤ 3p (11), up to one-third of the patients are disabled or dead after three months (12, 13).

Current American Heart Association/American Stroke Association guidelines indicate uncertainty regarding the use of intravenous thrombolysis (IVT) in patients with low NIHSS scores and non-disabling deficits (14), and recent European guidelines suggest against IVT in acute minor non-disabling stroke (15). Disabling strokes is not clearly defined in clinical practice, and excluding patients from acute treatment because their symptoms is considered too mild by the treating physician, might lead to unnecessary disability and deaths (16). A suggested definition is that a disabling deficit would prevent the patient from performing basic activities of daily living or returning to work (17), both clearly dependent on the premorbid function and age-related.

In light of the widespread clinical use of NIHSS in the acute phase defining stroke severity and the total NIHSS scores limitations, knowing how the individual NIHSS items relates to functional outcomes after three months is essential in both treatment guidance and prognostics. Since few stroke registers report the full NIHSS with sub-items, the evidence is limited. We aimed to study how the individual items of NIHSS at admission relates with an excellent outcome, modified Rankin Scale (mRS) ≤1p, three months post-stroke in an unselected stroke population.

Methods

Study design

This cross-sectional retrospective and register-based study used data from two Swedish stroke registers (Riksstroke, the national quality register for stroke care in Sweden (18) and Väststroke, a local stroke register in Gothenburg). The two registers provide different information about the same patients. The datasets were merged through personal identification numbers into one pseudonymized database.

Study sample

For peer review only - http://bmjopen.bmj.com/site/about/guidelines.xhtml

This study includes all the patients admitted with an acute stroke to the Sahlgrenska University Hospital from November 2014 to August 2018. The Sahlgrenska University Hospital consists of three sites, each with a comprehensive stroke unit, and provides emergency and basic care for 800,000 inhabitants in Gothenburg as well as specialized care for 1.8 million inhabitants in the west of Sweden. Patients matching the inclusion criteria were adults (≥ 18 years) with the diagnosis first ever ischemic stroke (I63) or intracerebral hemorrhage (I61) according to the International Classification of Diseases (ICD-10), and with available NIHSS items scores at admission, and mRS at three months follow-up. Patients with mRS = 6 (dead) where excluded.

Procedure and study variables

Väststroke contains information on the NIHSS at admission, assessed by physicians. In cases with missing NIHSS in the Väststroke register, assessments were retrieved from medical records when possible (19). In Sweden, stroke patients are invited to a three-month outpatient follow-up visit for a medical review and assessment of functional status (mRS). The data on mRS were collected from the charts or by telephone from the nurses in a structured way (20). Permanent institution residents are not invited to the follow-up. All variables in this study with the exception of mRS at 3 months were collected at the index stay. In Riksstroke the data were recorded by trained nurses working at the stroke units. Data retrieved from Riksstroke includes variables like sex, age, vascular risk factors, reperfusion treatment, and discharge destination. The explanatory variables where NIHSS items, and each NIHSS item component on admission was dichotomized into; no symptom (score 0) or symptom(s) (score of 1 or more). The outcome variable was mRS, and an excellent outcome was defined as mRS ≤1p.

Statistics

Descriptive demographic and clinical measures are given as mean ± SD, median and interquartile range (IQR) or as numbers and percentages as appropriate. Categorical variables were compared with chi-square ($\chi 2$) and Mann–Whitney U-test for continuous variables. Participants were divided into two groups based on mRS levels (mRS ≤ 1 indicating an excellent outcome or mRS > 1, indicating from slight to severe disability). In order to identify NIHSS items as predictors of an excellent outcome, logistic regression models were fitted and adjusted for the predefined variables such as age, sex, premorbid independency, stroke subtype, and admission stroke severity (according to NIHSS; minor 0-3, mild to moderate 4-15; severe ≥16). As an exploratory analysis, a second model stratified for the stroke subtypes, ischemic stroke or hemorrhage, and adjusted for age, sex, premorbid independency, stroke severity, and reperfusion therapy (in case of ischemic stroke), were built (supplementary table 2). The results are given in odds ratios (OR) with a 95% confidence interval (CI). The strength of relationship between the excellent outcome and NIHSS items was investigated using Spearman rho of correlation coefficient. Determining the strength of the relationship; Correlation coefficient 0.1-0.29 (small), 0.30-0.49 (medium), and 0.5-1.0 (strong). All statistical tests were two sided with alpha 5 %. The analyses were performed using IBM SPSS Statistics 28 software.

Patient and public involvement

There were no patient or members of the public involved in the planning of this study.

Ethics

For peer review only - http://bmjopen.bmj.com/site/about/guidelines.xhtml

The study was approved by the Regional Ethical Review Board in Gothenburg, Sweden on May 4, 2016, registration number 346-16, with an amendment approved on September 14, 2018, registration number T807-18. According to the Data Inspection Board in Sweden data from quality registers are exempt from the general rule of patient consent (the Personal Data Act, Swedish law No. SFS 1998:204), since their purpose is to improve the quality of care, treatment, and rehabilitation which is of public interest. Furthermore, the patients were informed that their data were reported to the stroke registers and could be used for research. The patients may opt-out with withdrawal of their data at any time. The data were pseudonymized, thus patients cannot be identified.

Results

Out of 3740 patients admitted with acute stroke, 1471 (39 %) patients had available data on NIHSS items at admission and mRS at three months follow-up; see the flow chart in Fig. 1. Among the 2269 not included patients, 374 (16.5 %) died during the hospital stay, and in general, they were older, with a mean age of 75 ± 13.5 years, and had higher mean NIHSS (6.9 ± 8) (Supplemental Table 1).

Of 1471 included patients, 48 % were female, the mean age was 72 ± 14.5 years, and with mean NIHSS 3.8 ± 5.1 at stroke admission. Median (IQR) mRS at 3 months was 2 (2). 91 % of the strokes were ischemic, and 66 % were considered mild with NIHSS ≤ 3. Facial palsy (in 31 % of the stroke cases), followed by dysarthria (30 %), was the most common scored NIHSS item. There were significantly higher proportion of patients with left- side than right-side motor symptoms ($p<0.001$). Patient characteristics and NIHSS items sub-scores are presented in Table 1. The distribution of mRS at three months based on stroke severity at admission is shown in Figure 2, where 26 % of patients with mild strokes had mRS ≤ 1 at three months.

In binary logistic crude regression models, scores in any NIHSS sub-items significantly reduced the odds of an excellent outcome (mRS ≤ 1) at three months. After adjustments for the other independent variables (age, sex, premorbid independency, stroke subtype, and stroke severity), scores on the right arm and leg motor symptoms and visual fields reduced the odds of an excellent outcome (mRS ≤ 1) at three months, OR 0.60 (95% CI: 0.37-0.98), OR 0.60 (95% CI: 0.37-0.97), and OR 0.65 (95% CI: 0.45-0.94), respectively (Figure 3). When stratifying for ischemic or hemorrhagic stroke, the results were comparable for ischemic stroke to those for the entire cohort, but no NIHSS item or side predicted outcome in hemorrhagic stroke. (Supplementary table 2).

When exploring the strength of relationship between dichotomized NIHSS items and excellent outcome three months after stroke (mRS ≤ 1), a small negative correlation was found between all the different NIHSS items and outcome. Orientation, language, and right leg motor had the highest, jet low, correlation, r=-0.202, r=-0.183, and r=-0.182, $p < 0.001$, respectively (Table 2).

Discussion

In this cross-sectional retrospective and register-based study, patients with first-ever stroke and scores on the NIHSS items right motor symptoms or visual field at admission where less likely to have excellent outcome at three months.

For peer review only - http://bmjopen.bmj.com/site/about/guidelines.xhtml

A possible explanation for the NIHSS right motor items importance for prognosis is the weighting of motor symptoms in the mRS and affection of dominant hemisphere. The mRS attempts to measure functional independence, incorporating the Word Health Organizations components of body function, activity, and participation, but domains such as cognition or language are not directly measured (21, 22). Abnormal motor items on the NIHSS in minor ischemic strokes (NIHSS <5) has been linked to poor outcome in two other studies, but none of them looked at the side of lesions (23, 24). When assessing stroke outcomes by the side of involvement, the evidence is inconclusive, although one might think that affecting language and dominant hand function would negatively influence the outcome (25, 26). Several scale items require intact language function, and studies have showed that patients with left-sided stroke and total anterior circulation syndromes score 4 points higher on the NIHSS than right-sided stroke patients despite smaller stroke volumes on imaging (6, 27). The weighting of NIHSS items associated with left-side lesions makes the NIHSS less suitable to predict outcome for right-side strokes (28). Our study, looking at dichotomized sub-items, supports the importance of dominant hemisphere affection.

The NIHSS item visual field reduces the odds of an excellent outcome in our study. Several studies have shown that visual field defects impact functional ability, quality of life, ability to participate in rehabilitation, and poor long-term recovery (29-34). Visual field defect could lead to loss of driving license, automatically giving mRS > 1, and linked to the risk of social isolation, depression and risk of requiring placement in long-term care (35). The most common type of visual field loss is homonymous hemianopia, most common in strokes affecting posterior circulation, supplying optic radiations, occipital cortex and other brain areas involved in visual field (36). NIHSS is generally less sensitive to strokes affecting posterior circulation, commonly causing vertigo and impaired balance (37), where a low NIHSS do not reflect stroke severity (9). In our cohort, 17.5 % had visual field defects, fewer than in many other studies (36, 38). The screening nature of vision testing in the acute setting and many patients being unaware of their loss might explain this (39). Our finding underpin the importance of visual field defects and testing in patients with otherwise low total NIHSS.

Our results suggest that in patients with right motor symptoms or visual field defects, IVT should not be withhold due to low NIHSS alone, in line with previous work (13). Both guidelines and study protocols describe minor or isolated non-disabling symptoms as reasons to withhold IVT, but no clear description exists. Suggested deficits are ataxia alone, sensory loss alone, dysarthria or mild aphasia alone, minimal weakness, isolated facial droop or mild cortical hand (14, 15, 17). As in our study, two other studies have shown that motor symptoms predict poor outcome, but instead of vision they report ataxia as a predictor (9, 23).

In general, and in line with previous studies, the individual NIHSS items explained only a small portion of the variance in outcome (23, 40, 41). Long-term follow-up with sensitive outcome measures is needed to make good prediction models, especially in minor strokes. When stratifying for ischemic or hemorrhagic stroke in a regression model, no NIHSS item or side predicted outcome in hemorrhagic stroke in adjusted models, probably due to the small and heterogenetic sample size.

The strength of this register-based study is a large, unselected stroke cohort, with a complete NIHSS score including sub-items and mRS at three months, collected in a real-life clinical setting, not excluding patients due to strict inclusion criteria. The overall coverage ratio in the Riksstroke register is 90 %, and taken together with the other factors mentioned above, the generalizability of our findings is substantial to countries with similar stroke population and health care system.

For peer review only - http://bmjopen.bmj.com/site/about/guidelines.xhtml

A limitation in our study is missing data, where 40 % of patients did not have a mRS assessed at three months. This reflect most clinical settings, with many patients lost for follow-up Another limitation is that we do not know the time from symptoms onset to NIHSS assessment, and studies have shown that NIHSS at 24 hours is better in prognostics, as many symptoms resolve during the first 24 hours (7). However, as our drop-out analyses show that patients excluded had larger strokes and higher mortality, our conclusions can contribute in the decision making process in acute treatment of minor stroke were selection to and effect of IVT are less established.

In conclusion, stroke patients with scores on the NIHSS items right motor symptoms or visual field at admission are less likely to have excellent outcome at three months. Clinicians should consider the NIHSS items affected, not only the total NIHSS score, both in treatment guidance and prognostics.

Author Contributors

G. Hagberg: drafting of the manuscript. M. Reinholdsson, Tamar Abzhandadze: acquisition of data. All authors contributed to study concept and design, analysis and interpretation of data and critical revision of the manuscript for intellectual content.

Data sharing statement

Data from registries are subject to the Personal Data Act (Swedish law No. SFS 1998:204). Data may be available to researchers upon request, after review of secrecy (contact the author ks.sunnerhagen@neuro.gu.se). According to the Swedish regulation (epn.se/en/start/regulations/), the permission to use data can only be according to application and approval from the ethical board.

Disclosure of interest

The authors declares that there is no conflict of interest regarding the publication of this paper.

Sources of funding

This work was supported by The Local Research and Development Board for Gothenburg and Södra Bohuslän, the Renée Eanders Foundation, the Swedish Stroke Association, the Swedish Heart-Lung Foundation, the Swedish Brain Foundation, Promobilia, and the Swedish Research Council VR 2017-00946. The Swedish state under the ALF agreement, an agreement between the Swedish government and county councils (ALFGBG-965653).

Acknowledgment

The authors thank the members of the Riksstroke Collaboration for data from the Swedish Stroke Register, Riksstroke (riksstroke.org), and the Väststroke Collaboration for data from the Väststroke Register. They also thank the participating patients and support staff for assistance with data acquisition.

For peer review only - http://bmjopen.bmj.com/site/about/guidelines.xhtml

References

1. Brott T, Adams HP, Jr., Olinger CP, et al. Measurements of acute cerebral infarction: a clinical examination scale. Stroke. 1989;20(7):864-70.
2. Goldstein LB, Bertels C, Davis JN. Interrater reliability of the NIH stroke scale. Archives of neurology. 1989;46(6):660-2.
3. Broderick JP, Lu M, Kothari R, et al. Finding the most powerful measures of the effectiveness of tissue plasminogen activator in the NINDS tPA stroke trial. Stroke. 2000;31(10):2335-41.
4. Adams HP, Jr., Davis PH, Leira EC, et al. Baseline NIH Stroke Scale score strongly predicts outcome after stroke: A report of the Trial of Org 10172 in Acute Stroke Treatment (TOAST). Neurology. 1999;53(1):126-31.
5. Finocchi C, Balestrino M, Malfatto L, et al. National Institutes of Health Stroke Scale in patients with primary intracerebral hemorrhage. Neurol Sci. 2018;39(10):1751-5.
6. Lyden P, Claesson L, Havstad S, et al. Factor analysis of the National Institutes of Health Stroke Scale in patients with large strokes. Arch Neurol. 2004;61(11):1677-80.
7. Abdul-Rahim AH, Fulton RL, Sucharew H, et al. National Institutes of Health Stroke Scale Item Profiles as Predictor of Patient Outcome: External Validation on Safe Implementation of Thrombolysis in Stroke-Monitoring Study Data. Stroke. 2015;46(10):2779-85.
8. Reznik ME, Yaghi S, Jayaraman MV, McTaggart RA, et al. Baseline NIH Stroke Scale is an inferior predictor of functional outcome in the era of acute stroke intervention. International journal of stroke : official journal of the International Stroke Society. 2018;13(8):806-10.
9. Sato S, Toyoda K, Uehara T, Toratani N, et al. Baseline NIH Stroke Scale Score predicting outcome in anterior and posterior circulation strokes. Neurology. 2008;70(24 Pt 2):2371-7.
10. Inoa V, Aron AW, Staff I, Fortunato G, Sansing LH. Lower NIH stroke scale scores are required to accurately predict a good prognosis in posterior circulation stroke. Cerebrovasc Dis. 2014;37(4):251-5.
11. Fischer U, Baumgartner A, Arnold M, Nedeltchev K, et al. What is a minor stroke? Stroke. 2010;41(4):661-6.
12. Khatri P, Conaway MR, Johnston KC. Ninety-day outcome rates of a prospective cohort of consecutive patients with mild ischemic stroke. Stroke. 2012;43(2):560-2.
13. Wendt M, Tütüncü S, Fiebach JB, et al. Preclusion of ischemic stroke patients from intravenous tissue plasminogen activator treatment for mild symptoms should not be based on low National Institutes of Health Stroke Scale Scores. J Stroke Cerebrovasc Dis. 2013;22(4):550-3.
14. Powers WJ, Rabinstein AA, Ackerson T, et al. 2018 Guidelines for the Early Management of Patients With Acute Ischemic Stroke: A Guideline for Healthcare Professionals From the American Heart Association/American Stroke Association. Stroke; a journal of cerebral circulation. 2018;49(3):e46-e110.
15. Berge E, Whiteley W, Audebert H, et al. European Stroke Organisation (ESO) guidelines on intravenous thrombolysis for acute ischaemic stroke. Eur Stroke J. 2021;6(1):I-lxii.
16. Barber PA, Zhang J, Demchuk AM, et al. Why are stroke patients excluded from TPA therapy? An analysis of patient eligibility. Neurology. 2001;56(8):1015-20.
17. Khatri P, Kleindorfer DO, Devlin T, et al. Effect of Alteplase vs Aspirin on Functional Outcome for Patients With Acute Ischemic Stroke and Minor Nondisabling Neurologic Deficits: The PRISMS Randomized Clinical Trial. Jama. 2018;320(2):156-66.
18. Asplund K, Hulter Åsberg K, Appelros P, et al. The Riks-Stroke story: building a sustainable national register for quality assessment of stroke care. International journal of stroke : official journal of the International Stroke Society. 2011;6(2):99-108.

For peer review only - http://bmjopen.bmj.com/site/about/guidelines.xhtml

19. Kasner SE, Cucchiara BL, McGarvey ML, et al. Modified National Institutes of Health Stroke Scale can be estimated from medical records. Stroke. 2003;34(2):568-70.
20. Bruno A, Akinwuntan AE, Lin C, et al. Simplified modified rankin scale questionnaire: reproducibility over the telephone and validation with quality of life. Stroke. 2011;42(8):2276-9.
21. van Swieten JC, Koudstaal PJ, Visser MC, et al. Interobserver agreement for the assessment of handicap in stroke patients. Stroke. 1988;19(5):604-7.
22. Kasner SE. Clinical interpretation and use of stroke scales. The Lancet Neurology. 2006;5(7):603-12.
23. Yaghi S, Willey JZ, Andrews H, Boehme AK, et al. The Itemized NIHSS Scores Are Associated With Discharge Disposition in Patients With Minor Stroke. Neurohospitalist. 2016;6(3):102-6.
24. Sato S, Uehara T, Ohara T, et al. Factors associated with unfavorable outcome in minor ischemic stroke. Neurology. 2014;83(2):174-81.
25. Di Legge S, Saposnik G, Nilanont Y, et al. Neglecting the difference: does right or left matter in stroke outcome after thrombolysis? Stroke. 2006;37(8):2066-9.
26. Fink JN, Frampton CM, Lyden P, et al.. Does hemispheric lateralization influence functional and cardiovascular outcomes after stroke?: an analysis of placebo-treated patients from prospective acute stroke trials. Stroke. 2008;39(12):3335-40.
27. Woo D, Broderick JP, Kothari RU, Lu M, et al. Does the National Institutes of Health Stroke Scale favor left hemisphere strokes? NINDS t-PA Stroke Study Group. Stroke. 1999;30(11):2355-9.
28. Yoo AJ, Romero J, Hakimelahi R, Nogueira RG, et al. Predictors of functional outcome vary by the hemisphere of involvement in major ischemic stroke treated with intra-arterial therapy: a retrospective cohort study. BMC Neurol. 2010;10:25.
29. Jones SA, Shinton RA. Improving outcome in stroke patients with visual problems. Age Ageing. 2006;35(6):560-5.
30. Leff AP, Scott SK, Crewes H, Hodgson TL, et al. Impaired reading in patients with right hemianopia. Ann Neurol. 2000;47(2):171-8.
31. Ramrattan RS, Wolfs RC, Panda-Jonas S, et al. Prevalence and causes of visual field loss in the elderly and associations with impairment in daily functioning: the Rotterdam Study. Arch Ophthalmol. 2001;119(12):1788-94.
32. West CG, Gildengorin G, Haegerstrom-Portnoy G, et al. Is vision function related to physical functional ability in older adults? J Am Geriatr Soc. 2002;50(1):136-45.
33. Sand KM, Wilhelmsen G, Naess H, et al. Vision problems in ischaemic stroke patients: effects on life quality and disability. Eur J Neurol. 2016;23 Suppl 1:1-7.
34. Johnson N, Nisar T, Criswell A, et al. Long-Term Disability Outcomes for Patients With Ischemic Stroke Presenting With Visual Deficits. J Neuroophthalmol. 2022;42(4):518-23.
35. Owsley C, McGwin G, Jr. Vision and driving. Vision Res. 2010;50(23):2348-61.
36. Rowe FJ, Wright D, Brand D, et al. A prospective profile of visual field loss following stroke: prevalence, type, rehabilitation, and outcome. Biomed Res Int. 2013;2013:719096.
37. Libman RB, Kwiatkowski TG, Hansen MD, et al. Differences between anterior and posterior circulation stroke in TOAST. Cerebrovasc Dis. 2001;11(4):311-6.
38. Gray CS, French JM, Bates D, et al. Recovery of visual fields in acute stroke: homonymous hemianopia associated with adverse prognosis. Age Ageing. 1989;18(6):419-21.
39. Falke P, Abela BM, Jr., Krakau CE, et al. High frequency of asymptomatic visual field defects in subjects with transient ischaemic attacks or minor strokes. J Intern Med. 1991;229(6):521-5.
40. Leira EC, Ludwig BR, Gurol ME, et al. The types of neurological deficits might not justify withholding treatment in patients with low total National Institutes of Health Stroke Scale scores. Stroke. 2012;43(3):782-6.
41. Choi JC, Kim BJ, Han MK, et al. Utility of Items of Baseline National Institutes of Health Stroke Scale as Predictors of Functional Outcomes at Three Months after Mild Ischemic Stroke. J Stroke Cerebrovasc Dis. 2017;26(6):1306-13.

For peer review only - http://bmjopen.bmj.com/site/about/guidelines.xhtml

Table 1: Characteristics of the study sample

**Total**	**1471**
**Age**	
Mean (SD)	71.8 (14.5)
Median (IQR)	74 (82-63)
**Sex (female) (%)**	658 (47.7)
**Stroke type (%)**	
Ischemic	1335 (90.8)
Hemorrhagic	136 (9.2)
**Reperfusion therapy (%)**	242 (16.5)
**NIHSS**	
Mean (SD)	3.8 (5.1)
Median (IQR)	2 (5-0)
**Stroke severity (%)**	
Mild (NIHSS≤3)	965 (65.6)
Mild to moderate	429 (29.2)
Severe (NIHSS>15)	77 (5.2)
**mRS 3 months**	
Median (IQR)	2 (3-1)
Excellent outcome (mRS≤1)	703 (47.8)
**Discharged to (%)**	
Home	1045 (71.0)
Premorbid independency* (%)	1169 (79.5)
**NIHSS sub score (%)**	
1a Consciousness	90 (6.1)
1b Orientation	311 (21.1)
1c Commands	110 (7.5)
2 Best Gaze	169 (11.5)
3 Visual field	257 (17.5)
4 Facial palsy	454 (30.9)
5 Motor arm	
Right	147 (10.0)
Left	211 (14.3)
6 Motor leg	
Right	159 (10.8)
Left	213 (14.5)
7 Ataxia	206 (14.0)
8 Sensory	350 (23.8)
9 Best Language	340 (23.1)
10 Dysarthria	438 (29.8)
11 Neglect	183 (12.4)
**Cardiovascular risk/comorbidity (%)**	
Atrial fibrillation	310 (21.1)
Diabetes	246 (16.7)
Smoking	171 (11.6)
On statins	300 (20.4)
Hypertension	720 (48.9)

*140 missing, SD standard deviation, IQR interquartile range, NIHSS=National Institutes of Health Stroke Scale, mRS=modified Rankin scale

For peer review only - http://bmjopen.bmj.com/site/about/guidelines.xhtml

Table 2. Exploring the strength of relationship between dichotomized NIHSS items and excellent outcome (mRS≤1)

		Outcome according to mRS			
N (%)		mRS>1 n (%)	mRS≤1 n (%)	χ2	Spearman rho (Correlation)
1a Consciousness					
Absence		690 (90.7)	683 (97.3)		
Symptoms		71 (9.3)	19 (2.7)	<0.001	-0.138
1b Orientation (0 ref.)					
Absence		538 (70.7)	611 (87.4)		
Symptoms		223 (29.3)	88 (12.6)	<0.001	-0.204
1c Commands					
Absence		671 (88.8)	677 (96.4)		
Symptoms		85 (11.2)	25 (3.6)	<0.001	-0.145
2 Best Gaze					
Absence		608 (82.8)	655 (93.8)		
Symptoms		126 (17.2)	43 (6.2)	<0.001	-0.171
3 Visual field					
Absence		545 (75.2)	615 (88.9)		
Symptoms		180 (24.8)	77 (11.1)	<0.001	-0.178
4 Facial palsy					
Absence		462 (62.3)	524 (75.1)		
Symptoms		280 (37.7)	174 (24.9)	<0.001	-0.138
5 Motor arm					
Right					
	Absence	643 (85.3)	665 (94.9)		
	Symptoms	111 (14.7)	36 (5.1)	<0.001	-0.159
Left					
	Absence	607 (80.2)	639 (91.3)		
	Symptoms	150 (19.8)	61 (8.7)	<0.001	-0.158
6 Motor leg					
Right					
	Absence	619 (83.4)	664 (94.9)		
	Symptoms	123 (16.5)	36 (5.1)	<0.001	-0.182
Left					
	Absence	595 (79.8)	637 (91.1)		
	Symptoms	151 (20.2)	62 (8.9)	<0.001	-0.160
7 Ataxia					
Absence		590 (81.6)	619 (89.5)		
Symptoms		133 (18.4)	73 (10.5)	<0.001	-0.111
8 Sensory					
Absence		509 (69.3)	556 (81.9)		
Symptoms		225 (30.7)	125 (18.1)	<0.001	-0.146
9 Best Language					
Absence		500 (68.5)	582 (84.1)		
Symptoms		230 (31.5)	110 (15.9)	<0.001	-0.183
10 Dysarthria					
Absence		448 (61.5)	539 (77.4)		
Symptoms		281 (38.5)	157 (22.6)	<0.001	-0.173
11 Neglect					
Absence		561 (81.4)	625 (91.9)		
Symptoms		128 (18.6)	55 (8.1)	<0.001	-0.154

For peer review only

Determining the strength of the relationship; Correlation coefficient 0.1-0.29 (small), 0.30-0.49 (medium) and 0.5-1.0 (large), mRS=modified Rankin scale. Each NIHSS item component on admission was dichotomized into; no symptoms (score 0) or symptoms (score of 1 or more).

Figure 1: Flow chart for study population

Figure 2: Stroke severity at baseline and mRS at 3 months.

NIHSS=National Institutes of Health Stroke Scale; mRS=modified Rankin scale

Figure 3: Odds ratio for excellent outcome (mRS≤1) with NIHSS items sub-score in logistic regression. *Unadjusted, †Adjusted for age, gender, premorbid independency, stroke subtype and stroke severity, OR=odds ratio, CI=confidence interval, NIHSS=National Institutes of Health Stroke Scale. Each NIHSS item component on admission was dichotomized into; no symptoms (score 0) or symptoms (score of 1 or more).

For peer review only

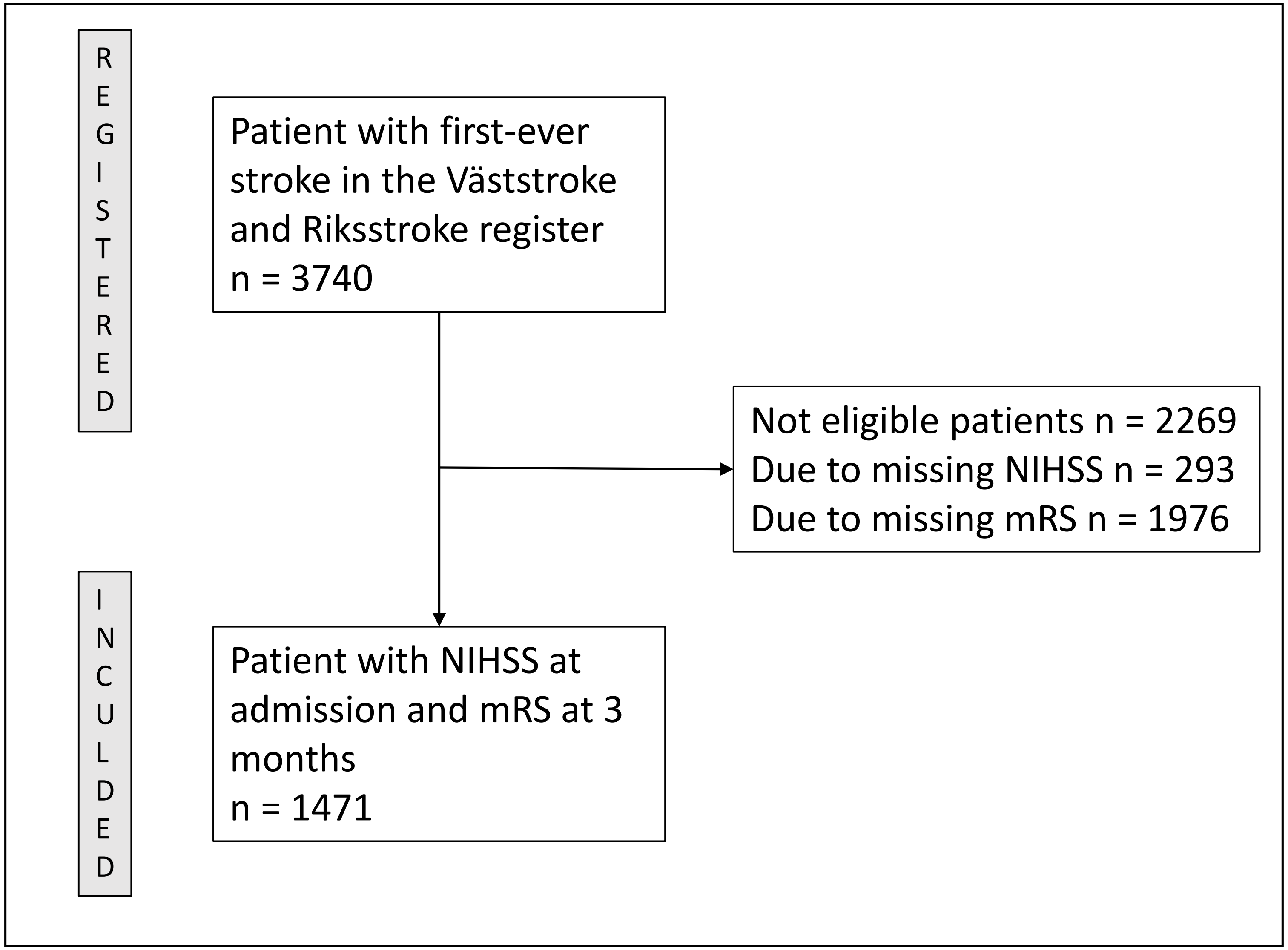
REGISTERED
Patient with first-ever stroke in the Väststroke and Riksstroke register
n = 3740
Not eligible patients n = 2269
Due to missing NIHSS n = 293
Due to missing mRS n = 1976
INCULDED
Patient with NIHSS at admission and mRS at 3 months
n = 1471

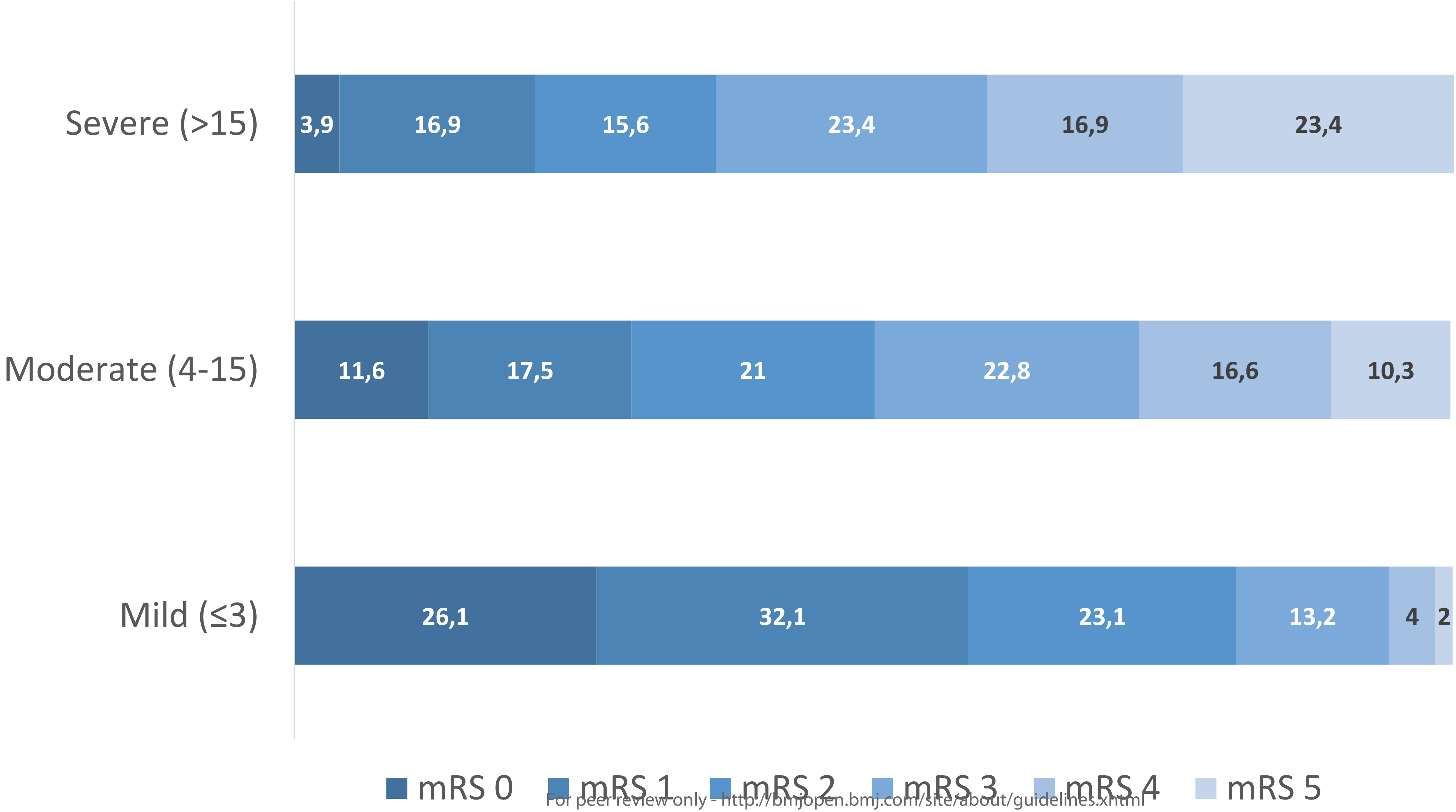
Severe (>15)
3,9
16,9
15,6
23,4
16,9
23,4
Moderate (4-15)
11,6
17,5
21
22,8
16,6
10,3
Mild (≤3)
26,1
32,1
23,1
13,2
4
2
mRS 0
mRS 1
mRS 2
mRS 3
mRS 4
mRS 5

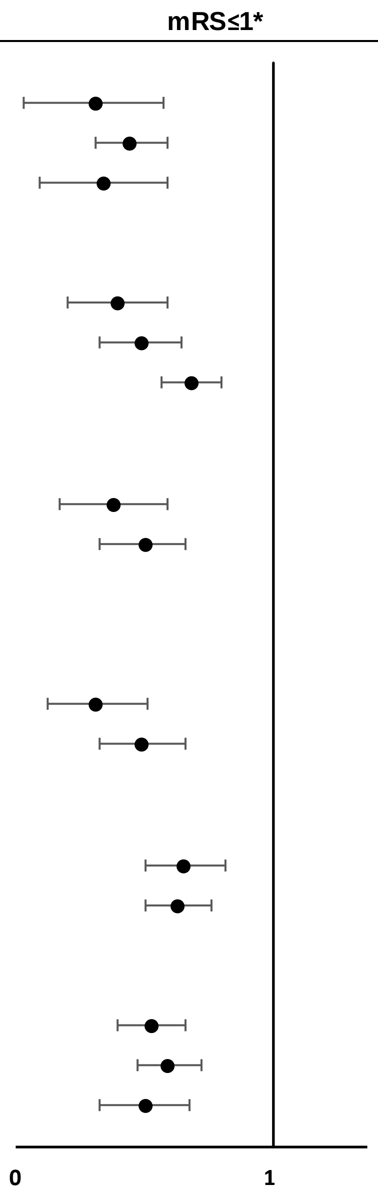

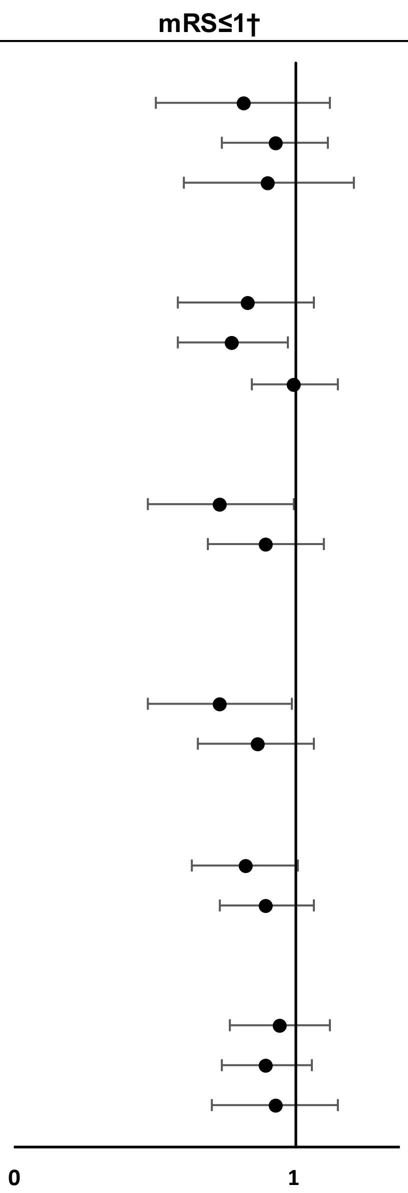

NIHSS sub items	OR (mRS≤1*)	95% CI		p-value	OR (mRS≤1†)	95% CI		p-value
1a Consciousness	0,27	0,16	0,45	<0,001	0,70	0,39	1,25	0,22
1b Orientation	0,35	0,27	0,46	<0,001	0,87	0,61	1,23	0,43
1c Commands	0,29	0,18	0,46	<0,001	0,83	0,47	1,48	0,53
2 Best Gaze	0,32	0,22	0,46	<0,001	0,72	0,45	1,13	0,15
3 Visual field	0,38	0,28	0,51	<0,001	0,65	0,45	0,94	0,02
4 Facial palsy	0,55	0,44	0,69	<0,001	0,99	0,74	1,32	0,93
5 Motor arm								
Right	0,31	0,21	0,46	<0,001	0,60	0,37	0,98	0,04
Left	0,39	0,28	0,53	<0,001	0,82	0,55	1,21	0,31
6 Motor leg								
Right	0,27	0,19	0,40	<0,001	0,60	0,37	0,97	0,04
Left	0,38	0,28	0,53	<0,001	0,77	0,52	1,13	0,18
7 Ataxia	0,52	0,39	0,71	<0,001	0,71	0,50	1,01	0,06
8 Sensory	0,50	0,39	0,64	<0,001	0,82	0,60	1,13	0,22
9 Best Language	0,41	0,32	0,53	<0,001	0,90	0,64	1,25	0,52
10 Dysarthria	0,46	0,37	0,59	<0,001	0,82	0,61	1,11	0,19
11 Neglect	0,39	0,28	0,54	<0,001	0,87	0,57	1,32	0,51

Supplementary table 1: Characteristics of patients not included

**Total**	**2269**
**Age**	
**Mean (SD)**	75 (14)*
**Median (IQR)**	77 (85-67)
**Sex (female) (%)**	1150 (51)*
**Stroke type (%)***	
**Ischemic**	1955 (86)
**Hemorrhagic**	350 (13)
**Reperfusion therapy (%)**	242 (16.5)
**NIHSS**	
**Mean (SD)**	7 (8)*
**Median (IQR)**	2 (8-0)
**Stroke severity (%)***	
**Mild (NIHSS≤3)**	1107 (49)
**Mild to moderate**	777 (34)
**Severe (NIHSS>15)**	380 (17)
**mRS 3 months (n = 293)**	
**Median (IQR)**	2 (3-1)
**Excellent outcome (mRS≤1)**	43 (15)*
**Discharged to (%)**	
**Home**	1252 (55)*
**Premorbid independency* (%)**	1169 (79.5)
**NIHSS sub score (%)**	
**1a Consciousness (n = 1382)**	125 (9)*
**1b Orientation (n = 1366)**	410 (30)*
**1c Commands (n = 1357)**	170 (13)*
**2 Best Gaze (n = 1329)**	240 (18)*
**3 Visual field (n = 1304)**	280 (21)*
**4 Facial palsy (n = 1332)**	454 (30.9)*
**5 Motor arm**	
**Right (n = 1354)**	213 (16)*
**Left (n = 1356)**	220 (16)
**6 Motor leg**	
**Right (n = 1348)**	222 (16)*
**Left (n = 1350)**	229 (17)*
**7 Ataxia (n = 1313)**	232 (18)
**8 Sensory (n = 1321)**	386 (29)*
**9 Best Language (n = 1317)**	422 (32)*
**10 Dysarthria (n = 1310)**	464 (35)*
**11 Neglect (n = 1256)**	194 (15)
**Cardiovascular risk/comorbidity (%)**	
**Atrial fibrillation**	450 (20)*
**Diabetes**	408 (18)
**Smoking**	291 (13)*
**On statins**	504 (22)
**Hypertension**	1210 (53)*

*signficant different from included cases. SD standard deviation, IQR interquartile range, NIHSS=National Institutes of Health Stroke Scale, mRS=modified Rankin scale

For peer review only

Supplementary table 2. Logistic regression; dichotomized NIHSS items sub-score stratified for stroke subtypes, and outcome mRS≤1

	Ischemic stroke				Hemorrhage			
	Crude		Adjusted§		Crude		Adjusted†	
NIHSS	OR	95% CI	OR	95% CI	OR	95% CI	OR	95% CI
1a Consciousness	0.29	0.17-0.51	0.70	0.37-1.30	0.21	0.05-0.97	0.83	0.13-5.30
1b Orientation (0 ref.)	0.34	0.25-0.45	0.83	0.58-1.21	0.52	0.23-1.18	1.42	0.48-4.18
1c Commands	0.31	0.19-0.50	0.90	0.49-1.66	0.21	0.05-0.99	0.31	0.03-2.76
2 Best Gaze	0.29	0.19-0.42	*0.57*	*0.34-0.94*	0.77	0.29-2.06	2.36	0.59-9.41
3 Visual field	*0.34*	*0.25-0.47*	*0.54*	*0.36-0.82*	0.97	0.44-2.16	1.54	0.55-4.30
4 Facial Palsy	0.55	0.43-0.69	0.89	0.66-1.22	0.59	0.27-1.29	1.81	0.59-5.57
5 Motor Arm								
Right	0.33	0.21-050	0.60	0.35-1.02	*0.32*	*0.11-0.90*	0.56	0.14-2.17
Left	0.38	0.27-0.53	0.73	0.48-1.11	0.53	0.22-1.31	1.19	0.37-3.85
6 Motor Leg								
Right	*0.27*	*0.18-0.41*	*0.58*	*0.35-0.97*	0.37	0.13-1.08	0.63	0.16-2.45
Left	0.38	0.27-0.54	0.69	0.45-1.05	0.50	0.21-1.17	0.95	0.30-3.08
7 Ataxia	0.49	0.36-0.68	0.71	0.49-1.03	0.94	0.35-2.59	1.01	0.30-3.38
8 Sensory	0.55	0.42-0.72	0.85	0.61-1.20	0.28	0.12-061	0.42	0.16-1.12
9 Best Language	0.43	0.33-0.57	0.95	0.66-1.36	0.30	0.13-0.70	0.58	0.21-1.60
10 Dysarthria	0.50	0.39-0.63	0.86	0.63-1.18	0.29	0.13-064	0.46	0.17-1.24
11 Neglect	0.36	0.25-0.52	0.75	0.47-1.19	0.69	0.29-1.64	1.83	0.57-5.83
Motor arm symptoms	0.31	0.24-0.42	*0.56*	*0.37-0.84*	0.30	0.14-0.64	0.60	0.19-1.91
Motor leg symptoms	0.30	0.23-0.40	*0.53*	*0.36-0.79*	0.33	0.15-0.72	0.60	0.18-1.94
Right motor symptoms	0.30	0.21-0.44	0.63	0.40-1.00	0.39	0.16-0.99	0.74	0.23-2.38
Left motor symptoms	0.40	0.29-0.54	0.69	0.48-1.00	0.51	0.22-1.16	0.90	0.29-2.76
Motor symptoms in at least one limb	0.32	0.25-0.41	0.55	0.38-0.79	0.32	0.15-0.68	0.63	0.20-2.05

§Adjusted for age, gender, premorbid independency, stroke severity and reperfusion therapy
† Adjusted for age, gender, premorbid independency and stroke severity
NIHSS=National Institutes of Health Stroke Scale, OR=odds ratio, CI=confidence interval
Each NIHSS item component on admission was dichotomized into; no symptoms (score 0) or symptoms (score of 1 or more). Significant values in Italic.

For peer review only

STROBE Statement—Checklist of items that should be included in reports of ***cohort studies***

	Item No	Recommendation	Page No
**Title and abstract**	1	(*a*) Indicate the study's design with a commonly used term in the title or the abstract	2
		(*b*) Provide in the abstract an informative and balanced summary of what was done and what was found	2
**Introduction**			
Background/rationale	2	Explain the scientific background and rationale for the investigation being reported	3
Objectives	3	State specific objectives, including any prespecified hypotheses	3
**Methods**			
Study design	4	Present key elements of study design early in the paper	3-4
Setting	5	Describe the setting, locations, and relevant dates, including periods of recruitment, exposure, follow-up, and data collection	3-4
Participants	6	(*a*) Give the eligibility criteria, and the sources and methods of selection of participants. Describe methods of follow-up	4
		(*b*) For matched studies, give matching criteria and number of exposed and unexposed	
Variables	7	Clearly define all outcomes, exposures, predictors, potential confounders, and effect modifiers. Give diagnostic criteria, if applicable	4
Data sources/ measurement	8*	For each variable of interest, give sources of data and details of methods of assessment (measurement). Describe comparability of assessment methods if there is more than one group	4
Bias	9	Describe any efforts to address potential sources of bias	
Study size	10	Explain how the study size was arrived at	4
Quantitative variables	11	Explain how quantitative variables were handled in the analyses. If applicable, describe which groupings were chosen and why	4
Statistical methods	12	(*a*) Describe all statistical methods, including those used to control for confounding	4
		(*b*) Describe any methods used to examine subgroups and interactions	
		(*c*) Explain how missing data were addressed	
		(*d*) If applicable, explain how loss to follow-up was addressed	
		(*e*) Describe any sensitivity analyses	
**Results**			
Participants	13*	(a) Report numbers of individuals at each stage of study—eg numbers potentially eligible, examined for eligibility, confirmed eligible, included in the study, completing follow-up, and analysed	5 Flow chart
		(b) Give reasons for non-participation at each stage	
		(c) Consider use of a flow diagram	
Descriptive data	14*	(a) Give characteristics of study participants (eg demographic, clinical, social) and information on exposures and potential confounders	5 Table1 Suppl.
		(b) Indicate number of participants with missing data for each variable of interest	
		(c) Summarise follow-up time (eg, average and total amount)	
Outcome data	15*	Report numbers of outcome events or summary measures over time	

For peer review only

For peer review only - http://bmjopen.bmj.com/site/about/guidelines.xhtml

Main results	16	(*a*) Give unadjusted estimates and, if applicable, confounder-adjusted estimates and their precision (eg, 95% confidence interval). Make clear which confounders were adjusted for and why they were included<br>(*b*) Report category boundaries when continuous variables were categorized<br>(*c*) If relevant, consider translating estimates of relative risk into absolute risk for a meaningful time period	5
Other analyses	17	Report other analyses done—eg analyses of subgroups and interactions, and sensitivity analyses	5<br>Suppl.
**Discussion**			
Key results	18	Summarise key results with reference to study objectives	5
Limitations	19	Discuss limitations of the study, taking into account sources of potential bias or imprecision. Discuss both direction and magnitude of any potential bias	6
Interpretation	20	Give a cautious overall interpretation of results considering objectives, limitations, multiplicity of analyses, results from similar studies, and other relevant evidence	7
Generalisability	21	Discuss the generalisability (external validity) of the study results	6
**Other information**			
Funding	22	Give the source of funding and the role of the funders for the present study and, if applicable, for the original study on which the present article is based	7

*Give information separately for exposed and unexposed groups.

Note: An Explanation and Elaboration article discusses each checklist item and gives methodological background and published examples of transparent reporting. The STROBE checklist is best used in conjunction with this article (freely available on the Web sites of PLoS Medicine at http://www.plosmedicine.org/, Annals of Internal Medicine at http://www.annals.org/, and Epidemiology at http://www.epidem.com/). Information on the STROBE Initiative is available at http://www.strobe-statement.org.

For peer review only

BMJ Open

A registry based study on the prognostic value of acute National Institutes of Health Stroke Scale Items on disability three months post-stroke

Journal:	*BMJ Open*
Manuscript ID	bmjopen-2023-080007.R1
Article Type:	Original research
Date Submitted by the Author:	07-Nov-2023
Complete List of Authors:	Hagberg, Guri; Oslo universitetssykehus Ullevål, Neurological Department ; University of Gothenburg Institute of Neuroscience and Physiology, Section for Clinical Neuroscience and Rehabilitation<br>Ihle-Hansen, Haakon; Vestre Viken HF, Department of medical research; University of Oslo, Institute of Clinical Medicine<br>Abzhandadze, Tamar; University of Gothenburg, Department of Clinical Neuroscience, Rehabilitation Medicine; Sahlgrenska University Hospital Department of Occupational Therapy and Physiotherapy<br>Reinholdsson, Malin; University of Gothenburg Institute of Neuroscience and Physiology, Rehabilitation medicine; Sahlgrenska University Hospital Department of Occupational Therapy and Physiotherapy,<br>Hansen, Hege; Vestre Viken HF, Department of Medicine; Oslo University Hospital, Department of Neurology<br>Sunnerhagen, Katharina; University of Gothenburg Institute of Neuroscience and Physiology, Institute of Neuroscience and Physiology
**Primary Subject Heading**:	Neurology
Secondary Subject Heading:	Emergency medicine, Medical management
Keywords:	Stroke < NEUROLOGY, Prognosis, Stroke medicine < INTERNAL MEDICINE, REGISTRIES

SCHOLARONE™
Manuscripts

I, the Submitting Author has the right to grant and does grant on behalf of all authors of the Work (as defined in the below author licence), an exclusive licence and/or a non-exclusive licence for contributions from authors who are: i) UK Crown employees; ii) where BMJ has agreed a CC-BY licence shall apply, and/or iii) in accordance with the terms applicable for US Federal Government officers or employees acting as part of their official duties; on a worldwide, perpetual, irrevocable, royalty-free basis to BMJ Publishing Group Ltd ("BMJ") its licensees and where the relevant Journal is co-owned by BMJ to the co-owners of the Journal, to publish the Work in this journal and any other BMJ products and to exploit all rights, as set out in our licence.

The Submitting Author accepts and understands that any supply made under these terms is made by BMJ to the Submitting Author unless you are acting as an employee on behalf of your employer or a postgraduate student of an affiliated institution which is paying any applicable article publishing charge ("APC") for Open Access articles. Where the Submitting Author wishes to make the Work available on an Open Access basis (and intends to pay the relevant APC), the terms of reuse of such Open Access shall be governed by a Creative Commons licence – details of these licences and which Creative Commons licence will apply to this Work are set out in our licence referred to above.

Other than as permitted in any relevant BMJ Author's Self Archiving Policies, I confirm this Work has not been accepted for publication elsewhere, is not being considered for publication elsewhere and does not duplicate material already published. I confirm all authors consent to publication of this Work and authorise the granting of this licence.

For peer review only

A registry based study on the prognostic value of acute National Institutes of Health Stroke Scale Items on disability three months post-stroke

MD PhD Guri Hagberg[1,3] MD PhD Haakon Ihle-Hansen[2], OT PhD Tamar Abzhandadze[3,4], PT MSc Malin Reinholdsson[3,4], MD PhD Hege Ihle-Hansen[1,2], Prof Katharina S. Sunnerhagen[3,5]

[1]Oslo Stroke Unit, Department of Neurology, Oslo University Hospital, Ullevål, Norway.

[2]Bærum Hospital- Vestre Viken Hospital Trust, Department of Medicine, Drammen, Norway.

[3]Institute of Neuroscience and Physiology, Section for Clinical Neuroscience and Rehabilitation, Gothenburg University, Gothenburg, Sweden

[4]Department of Occupational Therapy and Physiotherapy, Sahlgrenska University Hospital, Gothenburg, Sweden

[5]Neurocare, Sahlgrenska University Hospital, Gothenburg, Sweden.

Corresponding author: Guri Hagberg, Institute of Neuroscience and Physiology, Section for Clinical Neuroscience and Rehabilitation, Gothenburg University, Gothenburg, Sweden.

Mail: guri.hagberg@gmail.com

Running title: The prognostic value of NIHSS sub-items

Keywords: Stroke, NIHSS, mRS, prognosis

Word count: 2584

For peer review only

Abstract

Objectives We aimed to study how the individual items of National Institutes of Health Stroke Scale (NIHSS) at admission predicts functional independence three months post-stroke in patients with first-ever stroke.

Setting This registry-based study used data from two Swedish stroke registers (Riksstroke, the mandatory national quality register for stroke care in Sweden and Väststroke, a local quality stroke register in Gothenburg).

Participants This study included patients with first-ever acute stroke admitted from November 2014 to August 2018, with available NIHSS at admission and modified Rankin Scale (mRS) at three months follow-up.

Primary outcome The primary outcome variable was mRS ≤1 (defined as an excellent outcome) at three months follow-up.

Results We included 1471 patients, mean age was 72 (± 14.5) years, 48 % were female, and 66 % had mild strokes (NIHSS ≤ 3). In adjusted binary logistic regression analysis, the NIHSS items impaired right motor arm and leg, and impairment in visual field, reduced the odds of an excellent outcome at three months ((OR 0.60 (95% CI: 0.37-0.98), OR 0.60 (95% CI: 0.37-0.97), and OR 0.65 (95% CI: 0.45-0.94)).When exploring the effect size of associations between NIHSS items and mRS ≤1p, orientation, language, and right leg motor had the largest, yet small association.

Conclusions Stroke patients with scores on the NIHSS items right motor symptoms or visual field at admission are less likely to have excellent outcome at three months. Clinicians should consider the NIHSS items affected, not only the total NIHSS score, both in treatment guidance and prognostics.

Key words: Stroke, NIHSS, prognosis, mRS

Strengths and limitations of this study

- This study is based on registry data of high quality and high coverage, reflecting real-life clinical setting, potentially minimizing the risk of selection bias.
- Registry data have limitations with missing data that might limit the generalizability.
- To our knowledge, this study in the first to examine the prognostic merit of NIHSS individual items regardless of stroke severity on post-stroke functioning.

For peer review only - http://bmjopen.bmj.com/site/about/guidelines.xhtml

Introduction

The National Institutes of Health Stroke Scale (NIHSS) includes 15 individual items that measure motor and sensory function, vision, level of consciousness, attention, neglect, language, and speech production (1, 2). The NIHSS is the most widespread clinical scale used in patients presenting with acute stroke, and a high total NIHSS score is associated with poor outcomes in both hemorrhagic and ischemic strokes (3-5).

The total NIHSS score has several limitations as predictor of later stroke outcomes. The 15 items are weighted similarly, scoring 0-42, but left brain strokes score in general 4 points higher on the NIHSS than right brain strokes of larger volume (6). Total NIHSS scores at admission are inferior to 24 h and discharge scores in predicting post-stroke functional outcomes, due to spontaneous improvement and effect of acute interventions like thrombolysis or thrombectomy (7, 8). In strokes affecting posterior circulation, a low NIHSS might not predict a good functional outcome as the NIHSS is biased towards anterior circulation strokes (9, 10). In addition, in minor stroke, defined as NIHSS ≤ 3p (11), up to one-third of the patients are disabled or dead after three months (12, 13).

As the stroke field evolves, physicians tend to treat more small strokes with reperfusion therapy, although the published papers supporting this is lacking (14). Current American Heart Association/American Stroke Association guidelines indicate uncertainty regarding the use of intravenous thrombolysis (IVT) in patients with low NIHSS scores and non-disabling deficits (15), and recent European guidelines suggest against IVT in acute minor non-disabling stroke (16). Disabling strokes is not clearly defined in clinical practice, and excluding patients from acute treatment because their symptoms is considered too mild by the treating physician, might lead to unnecessary disability and deaths (17, 18). A suggested definition is that a disabling deficit would prevent the patient from performing basic activities of daily living or returning to work (19), both clearly dependent on the patients premorbid function and age.

In light of the widespread clinical use of NIHSS in the acute phase defining stroke severity and the total NIHSS scores limitations, knowing how the individual NIHSS items relates to functional outcomes after three months is essential in both treatment guidance and prognostics. Since few stroke registers report the full NIHSS with sub-items, the evidence on the prognostic merit of NIHSS individual items regardless of stroke severity on post-stroke functioning is lacking. We aimed to study how the individual items of NIHSS at admission predicts functional independence, modified Rankin Scale (mRS) ≤1, three months post-stroke in an unselected stroke population.

Methods

Study design

This register-based study used data from two Swedish stroke registers (Riksstroke, the mandatory national quality register for stroke care in Sweden (20) and Väststroke, a local quality stroke register in Gothenburg, collecting data from 2014 to 2019). The two registers provide different information about the same patients. The datasets were merged by a

For peer review only - http://bmjopen.bmj.com/site/about/guidelines.xhtml

statistician at Riksstroke through personal identification numbers into one pseudonymized database.

Study sample

This study includes all the patients ≥ 18 years admitted with a first-ever acute stroke (ischemic stroke (I63) or intracerebral hemorrhage (I61) according to the International Classification of Diseases (ICD-10), to the Sahlgrenska University Hospital from November 2014 to August 2018, with available NIHSS items scores at admission and mRS at three months follow-up. Further, permanent institution residents were not invited to the follow-up, and together with patients with mRS = 6 (dead) excluded from the study. The Sahlgrenska University Hospital consists of three sites, each with a comprehensive stroke unit, and provides emergency and basic care for 800,000 inhabitants in Gothenburg as well as specialized care for 1.8 million inhabitants in the west of Sweden.

Procedure and study variables

Väststroke contains information on the NIHSS at admission, assessed by physicians. In cases with missing NIHSS in the Väststroke register, assessments were retrieved from medical records when possible (21). In Sweden, stroke patients are invited to a three-month outpatient follow-up visit for a medical review and assessment of functional status (mRS). The data on mRS were collected from the charts or by telephone from the nurses in a structured way (22). All variables in this study with the exception of mRS at 3 months were collected at the index stay. In Riksstroke the data were recorded by trained nurses working at the stroke units. Data retrieved from Riksstroke includes variables like sex, age, vascular risk factors, reperfusion treatment, and discharge destination. The explanatory variables where NIHSS items, and each NIHSS item component on admission was dichotomized into; no symptom (score 0) or symptom(s) (score of 1 or more). The outcome variable was mRS, and an excellent outcome was defined as mRS ≤1.

Statistics

Descriptive demographic and clinical measures are given as mean ± SD, median and interquartile range (IQR) or as numbers and percentages as appropriate. Categorical variables were compared with chi-square ($\chi 2$) and Mann–Whitney U-test for continuous variables. Participants were divided into two groups based on mRS levels (mRS ≤ 1 indicating an excellent outcome or mRS > 1, indicating from slight to severe disability). In order to identify NIHSS items as predictors of an excellent outcome, logistic regression models were fitted and adjusted for the predefined variables such as age, sex, premorbid independency, stroke subtype, and admission stroke severity (according to NIHSS; minor 0-3, mild to moderate 4-15; severe ≥16). As an exploratory analysis, a second model stratified for the stroke subtypes, ischemic stroke or hemorrhage, and adjusted for age, sex, premorbid independency, stroke severity, and reperfusion therapy (in case of ischemic stroke), were built (Supplementary table 1). The results are given in odds ratios (OR) with a 95% confidence interval (CI). The effect size of the association between the excellent outcome and NIHSS items was investigated using phi coefficient; 0.1-0.29 (small effect), 0.30-0.49 (medium effect), and 0.5-1.0 (large effect) (23). All statistical tests were two sided with alpha 5 %. The analyses were performed using IBM SPSS Statistics 28 software.

For peer review only - http://bmjopen.bmj.com/site/about/guidelines.xhtml

Results

Out of 3740 patients admitted with acute stroke, 1471 (39 %) patients had available data on NIHSS items at admission and mRS at three months follow-up; see the flow chart in Figure 1. Among the 2269 not included patients, 374 (16.5 %) died during the hospital stay, and in general, they were older, with a mean age of 75 ± 13.5 years, and had higher mean NIHSS (6.9 ± 8) (Supplemental Table 2).

[insert figure 1]

Of 1471 included patients, 48 % were female, the mean age was 72 ± 14.5 years, and with mean NIHSS 3.8 ± 5.1 at stroke admission. Median (IQR) mRS at 3 months was 2 (2). Most (91 %) of the strokes were ischemic, and 66 % were considered mild with NIHSS ≤ 3. Facial palsy (in 31 % of the stroke cases), followed by dysarthria (30 %), was the most common scored NIHSS item. There were significantly higher proportion of patients with left side than right side motor symptoms ($p<0.001$). Patient characteristics and NIHSS items sub-scores are presented in Table 1. The distribution of mRS at three months based on stroke severity at admission is shown in Figure 2, where 26 % of patients with mild strokes had mRS ≤ 1 at three months.

[insert figure 2]

In binary logistic crude regression models, scores in any NIHSS sub-items significantly reduced the odds of an excellent outcome (mRS ≤ 1) at three months. After adjustments for the other independent variables (age, sex, premorbid independency, stroke subtype, and stroke severity), scores on the right arm and leg motor symptoms and visual fields reduced the odds of an excellent outcome (mRS ≤ 1) at three months, OR 0.60 (95% CI: 0.37-0.98), OR 0.60 (95% CI: 0.37-0.97), and OR 0.65 (95% CI: 0.45-0.94), respectively (Figure 3). When stratifying for ischemic or hemorrhagic stroke, the results were comparable for ischemic stroke to those for the entire cohort, but no NIHSS item or side predicted outcome in hemorrhagic stroke (Supplementary table 1).

[insert figure 3]

When exploring the effect size of association between dichotomized NIHSS items and excellent outcome three months after stroke (mRS ≤ 1), orientation, language, and right leg motor had the largest, jet small, association (0.202, 0.183, and 0.182, $p < 0.001$), respectively (Table 2).

Discussion

In this cross-sectional retrospective and register-based study, patients with first-ever stroke and scores on the NIHSS items right motor symptoms or visual field at admission where less likely to have excellent outcome at three months.

A possible explanation for the NIHSS right motor items importance for prognosis is the weighting of motor symptoms in the mRS and affection of dominant hemisphere. The mRS attempts to measure functional independence, incorporating the Word Health Organizations components of body function, activity, and participation, but domains such as cognition or language are not directly measured (24, 25). Abnormal motor items on the NIHSS in minor ischemic strokes (NIHSS <5) has been linked to poor outcome in two other studies, but none

For peer review only - http://bmjopen.bmj.com/site/about/guidelines.xhtml

of them looked at the side of lesions (26, 27). When assessing stroke outcomes by the side of involvement, the evidence is inconclusive, although one might think that affecting language and dominant hand function would negatively influence the outcome (28, 29). Several scale items require intact language function, and studies have showed that patients with left-sided stroke and total anterior circulation syndromes score 4 points higher on the NIHSS than right-sided stroke patients despite smaller stroke volumes on imaging (6, 30). The weighting of NIHSS items associated with left-side lesions makes the NIHSS less suitable to predict outcome for right-side strokes (31). Our study, looking at dichotomized sub-items, supports the importance of dominant hemisphere affection.

The NIHSS item visual field reduces the odds of an excellent outcome in our study. Several studies have shown that visual field defects impact functional ability, quality of life, ability to participate in rehabilitation, and poor long-term recovery (32-37). Visual field defect could lead to loss of driving license, automatically giving mRS > 1, and linked to the risk of social isolation, depression and risk of requiring placement in long-term care (38). The most common type of visual field loss is homonymous hemianopia, most common in strokes affecting posterior circulation, supplying optic radiations, occipital cortex and other brain areas involved in visual field (39). NIHSS is generally less sensitive to strokes affecting posterior circulation, commonly causing vertigo and impaired balance (40), where a low NIHSS do not reflect stroke severity (9). In our cohort, 17.5 % had visual field defects, fewer than in many other studies (39, 41). The screening nature of vision testing in the acute setting and many patients being unaware of their loss might explain this (42). Our finding underpin the importance of visual field defects and testing in patients with otherwise low total NIHSS.

Our results could suggest that in patients with right motor symptoms or visual field defects, IVT should not be withhold due to low NIHSS alone, in line with previous work (13). However, a recent published randomized clinical trial found that IVT was noninferior to dual anti platelet therapy regarding excellent functional outcome at 90 days in minor nondisabling acute ischemic stroke. They do not report on NIHSS items, but included patients with NIHSS ≤ 5, with less than or equal to 1 point on the sub items, including limb weakness or vision (14). Both guidelines and study protocols describe minor or isolated non-disabling symptoms as reasons to withhold IVT, but no clear description exists. Suggested deficits are ataxia alone, sensory loss alone, dysarthria or mild aphasia alone, minimal weakness, isolated facial droop or mild cortical hand (16, 19, 43). As in our study, two other studies have shown that motor symptoms predict poor outcome, but instead of vision they report ataxia as a predictor (9, 26), not supported in our data.

In general, and in line with previous studies, the individual NIHSS items explained only a small portion of the variance in outcome (26, 44, 45). Long-term follow-up with sensitive outcome measures is needed to make good prediction models, especially in minor strokes.

The strength of this register-based study is an unselected stroke cohort, with a complete NIHSS score including sub-items and mRS at three months, collected in a real-life clinical setting, not excluding patients due to strict inclusion criteria. The overall coverage ratio in the Riksstroke register is 90 % (46), and taken together with the other factors mentioned above, the generalizability of our findings to countries with similar stroke population and healthcare system seems likely.

A limitation in our study is missing data, where only 39 % had both available data on NIHSS items at admission and mRS at three months follow-up. However, as our drop-out analyses show that patients excluded had larger strokes and higher mortality, our conclusions can contribute in the decision making process in acute treatment of minor stroke were selection to

For peer review only - http://bmjopen.bmj.com/site/about/guidelines.xhtml

and effect of IVT are less established. Another limitation is that we do not know the time from symptoms onset to NIHSS assessment, and studies have shown that NIHSS at 24 hours is better in prognostics, as many symptoms resolve during the first 24 hours (7).

In conclusion, stroke patients with scores on the NIHSS items right motor symptoms or visual field at admission are less likely to have excellent outcome at three months. Clinicians should consider the NIHSS items affected, not only the total NIHSS score, both in treatment guidance and prognostics.

Patient and public involvement

There were no patient or members of the public involved in the planning of this study.

Ethics

The study was approved by the Regional Ethical Review Board in Gothenburg, Sweden on May 4, 2016, registration number 346-16, with an amendment approved on September 14, 2018, registration number T807-18. According to the Data Inspection Board in Sweden data from quality registers are exempt from the general rule of patient consent (the Personal Data Act, Swedish law No. SFS 1998:204), since their purpose is to improve the quality of care, treatment, and rehabilitation which is of public interest. Furthermore, the patients were informed that their data were reported to the stroke registers and could be used for research. The patients may opt-out with withdrawal of their data at any time. The data were pseudonymized, thus patients cannot be identified.

Author Contributors

G. Hagberg: drafting of the manuscript. M. Reinholdsson, T. Abzhandadze: acquisition of data. G. Hagberg, M. Reinholdsson, T. Abzhandadze, H. Ihle-Hansen, H. Ihle-Hansen and K.S. Sunnerhagen contributed to study concept and design, analysis and interpretation of data and critical revision of the manuscript for intellectual content.

Data sharing statement

Data from registries are subject to the Personal Data Act (Swedish law No. SFS 1998:204). Data may be available to researchers upon request, after review of secrecy (contact the author ks.sunnerhagen@neuro.gu.se). According to the Swedish regulation (epn.se/en/start/regulations/), the permission to use data can only be according to application and approval from the ethical board.

Disclosure of interest

The authors declares that there is no conflict of interest regarding the publication of this paper.

Sources of funding

This work was supported by The Local Research and Development Board for Gothenburg and Södra Bohuslän, the Renée Eanders Foundation, the Swedish Stroke Association, the Swedish Heart-Lung Foundation, the Swedish Brain Foundation, Promobilia, and the Swedish Research Council VR 2017-00946. The Swedish state under the ALF agreement, an agreement between the Swedish government and county councils (ALFGBG-965653).

For peer review only - http://bmjopen.bmj.com/site/about/guidelines.xhtml

For peer review only

Acknowledgment

The authors thank the members of the Riksstroke Collaboration for data from the Swedish Stroke Register, Riksstroke (riksstroke.org), and the Väststroke Collaboration for data from the Väststroke Register. They also thank the participating patients and support staff for assistance with data acquisition.

References

1. Brott T, Adams HP, Jr., Olinger CP, Marler JR, Barsan WG, Biller J, et al. Measurements of acute cerebral infarction: a clinical examination scale. Stroke. 1989;20(7):864-70.
2. Goldstein LB, Bertels C, Davis JN. Interrater reliability of the NIH stroke scale. Archives of neurology. 1989;46(6):660-2.
3. Broderick JP, Lu M, Kothari R, Levine SR, Lyden PD, Haley EC, et al. Finding the most powerful measures of the effectiveness of tissue plasminogen activator in the NINDS tPA stroke trial. Stroke. 2000;31(10):2335-41.
4. Adams HP, Jr., Davis PH, Leira EC, Chang KC, Bendixen BH, Clarke WR, et al. Baseline NIH Stroke Scale score strongly predicts outcome after stroke: A report of the Trial of Org 10172 in Acute Stroke Treatment (TOAST). Neurology. 1999;53(1):126-31.
5. Finocchi C, Balestrino M, Malfatto L, Mancardi G, Serrati C, Gandolfo C. National Institutes of Health Stroke Scale in patients with primary intracerebral hemorrhage. Neurol Sci. 2018;39(10):1751-5.
6. Lyden P, Claesson L, Havstad S, Ashwood T, Lu M. Factor analysis of the National Institutes of Health Stroke Scale in patients with large strokes. Arch Neurol. 2004;61(11):1677-80.
7. Abdul-Rahim AH, Fulton RL, Sucharew H, Kleindorfer D, Khatri P, Broderick JP, et al. National Institutes of Health Stroke Scale Item Profiles as Predictor of Patient Outcome: External Validation on Safe Implementation of Thrombolysis in Stroke-Monitoring Study Data. Stroke. 2015;46(10):2779-85.
8. Reznik ME, Yaghi S, Jayaraman MV, McTaggart RA, Hemendinger M, Mac Grory BC, et al. Baseline NIH Stroke Scale is an inferior predictor of functional outcome in the era of acute stroke intervention. International journal of stroke : official journal of the International Stroke Society. 2018;13(8):806-10.
9. Sato S, Toyoda K, Uehara T, Toratani N, Yokota C, Moriwaki H, et al. Baseline NIH Stroke Scale Score predicting outcome in anterior and posterior circulation strokes. Neurology. 2008;70(24 Pt 2):2371-7.
10. Inoa V, Aron AW, Staff I, Fortunato G, Sansing LH. Lower NIH stroke scale scores are required to accurately predict a good prognosis in posterior circulation stroke. Cerebrovasc Dis. 2014;37(4):251-5.
11. Fischer U, Baumgartner A, Arnold M, Nedeltchev K, Gralla J, De Marchis GM, et al. What is a minor stroke? Stroke. 2010;41(4):661-6.
12. Khatri P, Conaway MR, Johnston KC. Ninety-day outcome rates of a prospective cohort of consecutive patients with mild ischemic stroke. Stroke. 2012;43(2):560-2.
13. Wendt M, Tütüncü S, Fiebach JB, Scheitz JF, Audebert HJ, Nolte CH. Preclusion of ischemic stroke patients from intravenous tissue plasminogen activator treatment for mild symptoms should not be based on low National Institutes of Health Stroke Scale Scores. J Stroke Cerebrovasc Dis. 2013;22(4):550-3.
14. Chen HS, Cui Y, Zhou ZH, Zhang H, Wang LX, Wang WZ, et al. Dual Antiplatelet Therapy vs Alteplase for Patients With Minor Nondisabling Acute Ischemic Stroke: The ARAMIS Randomized Clinical Trial. Jama. 2023;329(24):2135-44.
15. Powers WJ, Rabinstein AA, Ackerson T, Adeoye OM, Bambakidis NC, Becker K, et al. Guidelines for the Early Management of Patients With Acute Ischemic Stroke: 2019 Update to the

For peer review only - http://bmjopen.bmj.com/site/about/guidelines.xhtml

2018 Guidelines for the Early Management of Acute Ischemic Stroke: A Guideline for Healthcare Professionals From the American Heart Association/American Stroke Association. Stroke; a journal of cerebral circulation. 2019;50(12):e344-e418.
16. Berge E, Whiteley W, Audebert H, De Marchis GM, Fonseca AC, Padiglioni C, et al. European Stroke Organisation (ESO) guidelines on intravenous thrombolysis for acute ischaemic stroke. Eur Stroke J. 2021;6(1):I-lxii.
17. Barber PA, Zhang J, Demchuk AM, Hill MD, Buchan AM. Why are stroke patients excluded from TPA therapy? An analysis of patient eligibility. Neurology. 2001;56(8):1015-20.
18. Duan C, Xiong Y, Gu HQ, Wang S, Yang KX, Hao M, et al. Outcomes in minor stroke patients treated with intravenous thrombolysis. CNS Neurosci Ther. 2023;29(8):2308-17.
19. Khatri P, Kleindorfer DO, Devlin T, Sawyer RN, Jr., Starr M, Mejilla J, et al. Effect of Alteplase vs Aspirin on Functional Outcome for Patients With Acute Ischemic Stroke and Minor Nondisabling Neurologic Deficits: The PRISMS Randomized Clinical Trial. Jama. 2018;320(2):156-66.
20. Asplund K, Hulter Åsberg K, Appelros P, Bjarne D, Eriksson M, Johansson A, et al. The Riks-Stroke story: building a sustainable national register for quality assessment of stroke care. International journal of stroke : official journal of the International Stroke Society. 2011;6(2):99-108.
21. Kasner SE, Cucchiara BL, McGarvey ML, Luciano JM, Liebeskind DS, Chalela JA. Modified National Institutes of Health Stroke Scale can be estimated from medical records. Stroke; a journal of cerebral circulation. 2003;34(2):568-70.
22. Bruno A, Akinwuntan AE, Lin C, Close B, Davis K, Baute V, et al. Simplified modified rankin scale questionnaire: reproducibility over the telephone and validation with quality of life. Stroke. 2011;42(8):2276-9.
23. Cohen J. A power primer. Psychol Bull. 1992;112(1):155-9.
24. van Swieten JC, Koudstaal PJ, Visser MC, Schouten HJ, van Gijn J. Interobserver agreement for the assessment of handicap in stroke patients. Stroke. 1988;19(5):604-7.
25. Kasner SE. Clinical interpretation and use of stroke scales. The Lancet Neurology. 2006;5(7):603-12.
26. Yaghi S, Willey JZ, Andrews H, Boehme AK, Marshall RS, Boden-Albala B. The Itemized NIHSS Scores Are Associated With Discharge Disposition in Patients With Minor Stroke. Neurohospitalist. 2016;6(3):102-6.
27. Sato S, Uehara T, Ohara T, Suzuki R, Toyoda K, Minematsu K. Factors associated with unfavorable outcome in minor ischemic stroke. Neurology. 2014;83(2):174-81.
28. Di Legge S, Saposnik G, Nilanont Y, Hachinski V. Neglecting the difference: does right or left matter in stroke outcome after thrombolysis? Stroke. 2006;37(8):2066-9.
29. Fink JN, Frampton CM, Lyden P, Lees KR. Does hemispheric lateralization influence functional and cardiovascular outcomes after stroke?: an analysis of placebo-treated patients from prospective acute stroke trials. Stroke. 2008;39(12):3335-40.
30. Woo D, Broderick JP, Kothari RU, Lu M, Brott T, Lyden PD, et al. Does the National Institutes of Health Stroke Scale favor left hemisphere strokes? NINDS t-PA Stroke Study Group. Stroke. 1999;30(11):2355-9.
31. Yoo AJ, Romero J, Hakimelahi R, Nogueira RG, Rabinov JD, Pryor JC, et al. Predictors of functional outcome vary by the hemisphere of involvement in major ischemic stroke treated with intra-arterial therapy: a retrospective cohort study. BMC Neurol. 2010;10:25.
32. Jones SA, Shinton RA. Improving outcome in stroke patients with visual problems. Age Ageing. 2006;35(6):560-5.
33. Leff AP, Scott SK, Crewes H, Hodgson TL, Cowey A, Howard D, et al. Impaired reading in patients with right hemianopia. Ann Neurol. 2000;47(2):171-8.
34. Ramrattan RS, Wolfs RC, Panda-Jonas S, Jonas JB, Bakker D, Pols HA, et al. Prevalence and causes of visual field loss in the elderly and associations with impairment in daily functioning: the Rotterdam Study. Arch Ophthalmol. 2001;119(12):1788-94.
35. West CG, Gildengorin G, Haegerstrom-Portnoy G, Schneck ME, Lott L, Brabyn JA. Is vision function related to physical functional ability in older adults? J Am Geriatr Soc. 2002;50(1):136-45.

For peer review only - http://bmjopen.bmj.com/site/about/guidelines.xhtml

36. Sand KM, Wilhelmsen G, Naess H, Midelfart A, Thomassen L, Hoff JM. Vision problems in ischaemic stroke patients: effects on life quality and disability. Eur J Neurol. 2016;23 Suppl 1:1-7.
37. Johnson N, Nisar T, Criswell A, McCane D, Lee J, Chiu D, et al. Long-Term Disability Outcomes for Patients With Ischemic Stroke Presenting With Visual Deficits. J Neuroophthalmol. 2022;42(4):518-23.
38. Owsley C, McGwin G, Jr. Vision and driving. Vision Res. 2010;50(23):2348-61.
39. Rowe FJ, Wright D, Brand D, Jackson C, Harrison S, Maan T, et al. A prospective profile of visual field loss following stroke: prevalence, type, rehabilitation, and outcome. Biomed Res Int. 2013;2013:719096.
40. Libman RB, Kwiatkowski TG, Hansen MD, Clarke WR, Woolson RF, Adams HP. Differences between anterior and posterior circulation stroke in TOAST. Cerebrovasc Dis. 2001;11(4):311-6.
41. Gray CS, French JM, Bates D, Cartlidge NE, Venables GS, James OF. Recovery of visual fields in acute stroke: homonymous hemianopia associated with adverse prognosis. Age Ageing. 1989;18(6):419-21.
42. Falke P, Abela BM, Jr., Krakau CE, Lilja B, Lindgärde F, Maly P, et al. High frequency of asymptomatic visual field defects in subjects with transient ischaemic attacks or minor strokes. J Intern Med. 1991;229(6):521-5.
43. Powers WJ, Rabinstein AA, Ackerson T, Adeoye OM, Bambakidis NC, Becker K, et al. 2018 Guidelines for the Early Management of Patients With Acute Ischemic Stroke: A Guideline for Healthcare Professionals From the American Heart Association/American Stroke Association. Stroke; a journal of cerebral circulation. 2018;49(3):e46-e110.
44. Leira EC, Ludwig BR, Gurol ME, Torner JC, Adams HP, Jr. The types of neurological deficits might not justify withholding treatment in patients with low total National Institutes of Health Stroke Scale scores. Stroke. 2012;43(3):782-6.
45. Choi JC, Kim BJ, Han MK, Lee SJ, Kang K, Park JM, et al. Utility of Items of Baseline National Institutes of Health Stroke Scale as Predictors of Functional Outcomes at Three Months after Mild Ischemic Stroke. J Stroke Cerebrovasc Dis. 2017;26(6):1306-13.
46. Söderholm A, Stegmayr B, Glader EL, Asplund K. Validation of Hospital Performance Measures of Acute Stroke Care Quality. Riksstroke, the Swedish Stroke Register. Neuroepidemiology. 2016;46(4):229-34.

For peer review only

For peer review only - http://bmjopen.bmj.com/site/about/guidelines.xhtml

Table 1: Characteristics of the study sample

**Total**	**1471**
**Age**	
Mean (SD)	71.8 (14.5)
Median (IQR)	74 (82-63)
**Sex (female) (%)**	658 (47.7)
**Stroke type (%)**	
Ischemic	1335 (90.8)
Hemorrhagic	136 (9.2)
**Reperfusion therapy (%)**	242 (16.5)
**NIHSS**	
Mean (SD)	3.8 (5.1)
Median (IQR)	2 (5-0)
**Stroke severity (%)**	
Mild (NIHSS≤3)	965 (65.6)
Mild to moderate	429 (29.2)
Severe (NIHSS>15)	77 (5.2)
**mRS 3 months**	
Median (IQR)	2 (3-1)
Excellent outcome (mRS≤1)	703 (47.8)
**Discharged to (%)**	
Home	1045 (71.0)
Premorbid independency* (%)	1169 (79.5)
**NIHSS sub score (% of 1471)**	
1a Consciousness	90 (6.1)
1b Orientation	311 (21.1)
1c Commands	110 (7.5)
2 Best Gaze	169 (11.5)
3 Visual field	257 (17.5)
4 Facial palsy	454 (30.9)
5 Motor arm	
Right	147 (10.0)
Left	211 (14.3)
6 Motor leg	
Right	159 (10.8)
Left	213 (14.5)
7 Ataxia	206 (14.0)
8 Sensory	350 (23.8)
9 Best Language	340 (23.1)
10 Dysarthria	438 (29.8)
11 Neglect	183 (12.4)
**Cardiovascular risk/comorbidity (%)**	
Atrial fibrillation	310 (21.1)
Diabetes	246 (16.7)
Smoking	171 (11.6)
On statins	300 (20.4)
Hypertension	720 (48.9)

*140 missing, SD standard deviation, IQR interquartile range, NIHSS=National Institutes of Health Stroke Scale, mRS=modified Rankin scale

For peer review only

For peer review only - http://bmjopen.bmj.com/site/about/guidelines.xhtml

Table 2. Exploring the strength of relationship between dichotomized NIHSS items and excellent outcome (mRS≤1)

N (%)		Outcome according to mRS<br>mRS>1<br>n (%)	mRS≤1<br>n (%)	χ2	Phi coefficient
1a Consciousness					
Absence		690 (90.7)	683 (97.3)		
Symptoms		71 (9.3)	19 (2.7)	<0.001	0.138
1b Orientation (0 ref.)					
Absence		538 (70.7)	611 (87.4)		
Symptoms		223 (29.3)	88 (12.6)	<0.001	0.204
1c Commands					
Absence		671 (88.8)	677 (96.4)		
Symptoms		85 (11.2)	25 (3.6)	<0.001	0.145
2 Best Gaze					
Absence		608 (82.8)	655 (93.8)		
Symptoms		126 (17.2)	43 (6.2)	<0.001	0.171
3 Visual field					
Absence		545 (75.2)	615 (88.9)		
Symptoms		180 (24.8)	77 (11.1)	<0.001	0.178
4 Facial palsy					
Absence		462 (62.3)	524 (75.1)		
Symptoms		280 (37.7)	174 (24.9)	<0.001	0.138
5 Motor arm					
Right					
	Absence	643 (85.3)	665 (94.9)		
	Symptoms	111 (14.7)	36 (5.1)	<0.001	0.159
Left					
	Absence	607 (80.2)	639 (91.3)		
	Symptoms	150 (19.8)	61 (8.7)	<0.001	0.158
6 Motor leg					
Right					
	Absence	619 (83.4)	664 (94.9)		
	Symptoms	123 (16.5)	36 (5.1)	<0.001	0.182
Left					
	Absence	595 (79.8)	637 (91.1)		
	Symptoms	151 (20.2)	62 (8.9)	<0.001	0.160
7 Ataxia					
Absence		590 (81.6)	619 (89.5)		
Symptoms		133 (18.4)	73 (10.5)	<0.001	0.111
8 Sensory					
Absence		509 (69.3)	556 (81.9)		
Symptoms		225 (30.7)	125 (18.1)	<0.001	0.146
9 Best Language					
Absence		500 (68.5)	582 (84.1)		
Symptoms		230 (31.5)	110 (15.9)	<0.001	0.183
10 Dysarthria					
Absence		448 (61.5)	539 (77.4)		
Symptoms		281 (38.5)	157 (22.6)	<0.001	0.173
11 Neglect					
Absence		561 (81.4)	625 (91.9)		
Symptoms		128 (18.6)	55 (8.1)	<0.001	0.154

Determining the effect size of the associations; Effect size 0.1-0.29 (small), 0.30-0.49 (medium) and 0.5-1.0 (large), mRS=modified Rankin scale. Each NIHSS item component on admission was dichotomized into; no symptoms (score 0) or symptoms (score of 1 or more).

For peer review only - http://bmjopen.bmj.com/site/about/guidelines.xhtml

Figure 1: Flow chart for study population

Figure 2: Stroke severity at baseline and mRS at 3 months

Percentage of patients with different mRS according to stroke severity (NIHSS; minor ≤ 3, mild to moderate 4-15; severe >15). NIHSS=National Institutes of Health Stroke Scale; mRS=modified Rankin scale.

Figure 3: Odds ratio for excellent outcome (mRS≤1) with NIHSS items sub-score in logistic regression (N = 1471)

*Unadjusted, †Adjusted for age, gender, premorbid independency, stroke subtype and stroke severity, OR=odds ratio, CI=confidence interval, NIHSS=National Institutes of Health Stroke Scale. Each NIHSS item component on admission was dichotomized into; no symptoms (score 0) or symptoms (score of 1 or more).

For peer review only

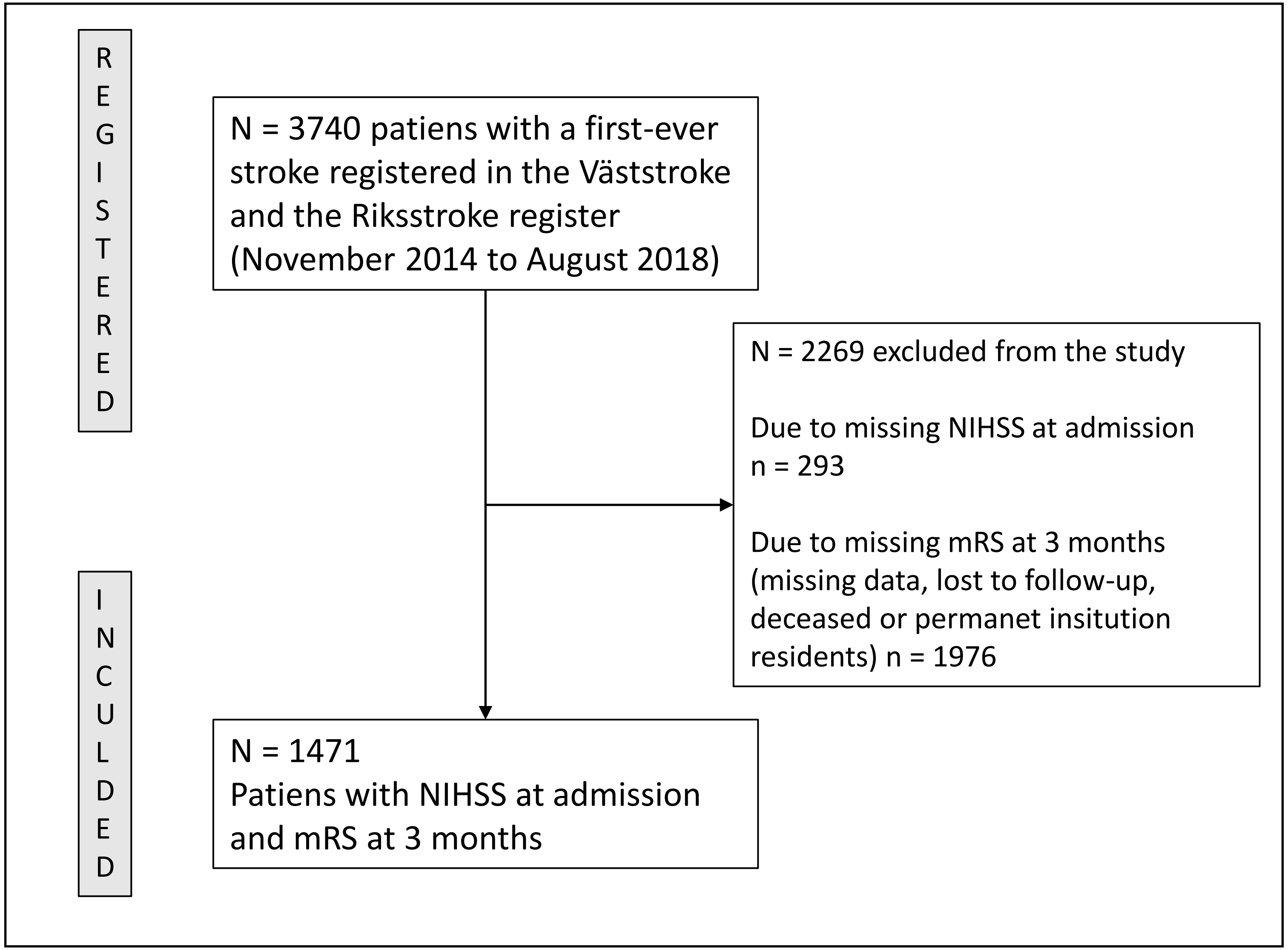
REGISTERED
N = 3740 patiens with a first-ever stroke registered in the Väststroke and the Riksstroke register (November 2014 to August 2018)
N = 2269 excluded from the study
Due to missing NIHSS at admission n = 293
Due to missing mRS at 3 months (missing data, lost to follow-up, deceased or permanet insitution residents) n = 1976
INCULDED
N = 1471
Patiens with NIHSS at admission and mRS at 3 months

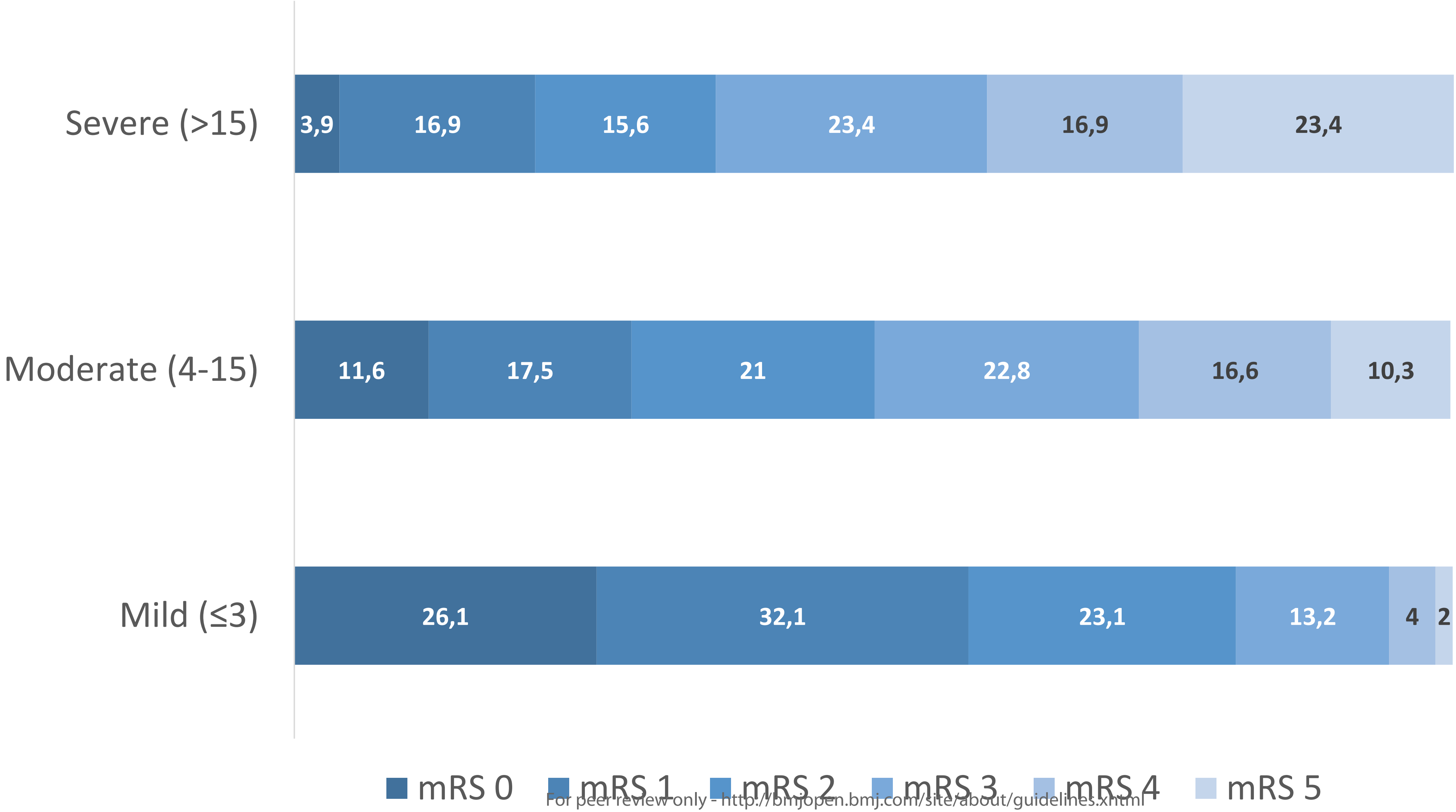
Severe (>15)
3,9
16,9
15,6
23,4
16,9
23,4
Moderate (4-15)
11,6
17,5
21
22,8
16,6
10,3
Mild (≤3)
26,1
32,1
23,1
13,2
4
2
mRS 0
mRS 1
mRS 2
mRS 3
mRS 4
mRS 5

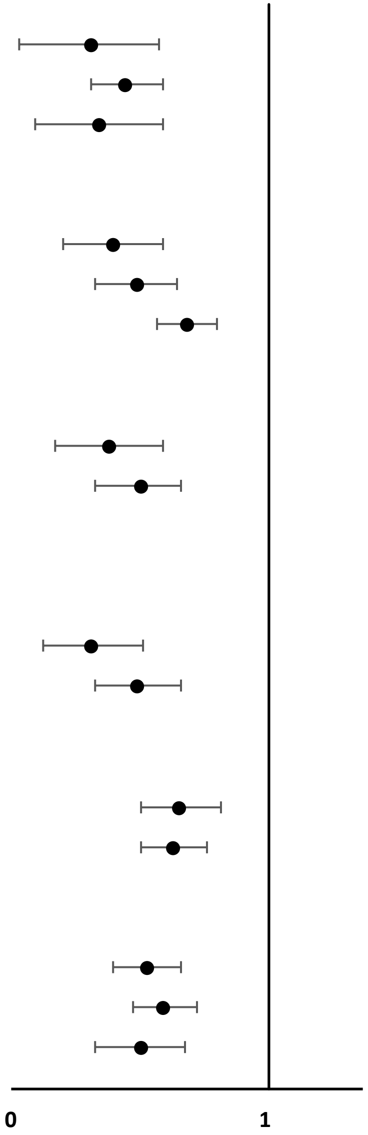

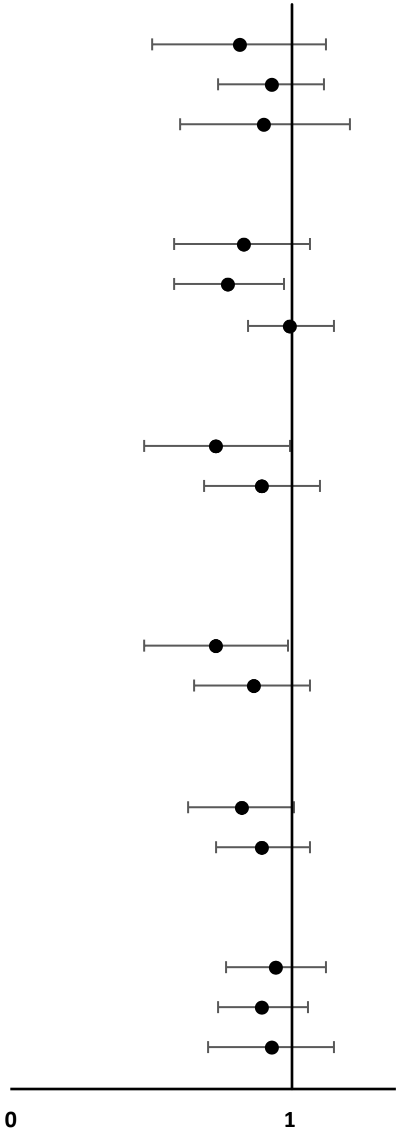

NIHSS sub items	mRS≤1* OR	95% CI		p-value	mRS≤1† OR	95% CI		p-value
1a Consciousness	0,27	0,16	0,45	<0,001	0,70	0,39	1,25	0,22
1b Orientation	0,35	0,27	0,46	<0,001	0,87	0,61	1,23	0,43
1c Commands	0,29	0,18	0,46	<0,001	0,83	0,47	1,48	0,53
2 Best Gaze	0,32	0,22	0,46	<0,001	0,72	0,45	1,13	0,15
3 Visual field	0,38	0,28	0,51	<0,001	0,65	0,45	0,94	0,02
4 Facial palsy	0,55	0,44	0,69	<0,001	0,99	0,74	1,32	0,93
5 Motor arm								
Right	0,31	0,21	0,46	<0,001	0,60	0,37	0,98	0,04
Left	0,39	0,28	0,53	<0,001	0,82	0,55	1,21	0,31
6 Motor leg								
Right	0,27	0,19	0,40	<0,001	0,60	0,37	0,97	0,04
Left	0,38	0,28	0,53	<0,001	0,77	0,52	1,13	0,18
7 Ataxia	0,52	0,39	0,71	<0,001	0,71	0,50	1,01	0,06
8 Sensory	0,50	0,39	0,64	<0,001	0,82	0,60	1,13	0,22
9 Best Language	0,41	0,32	0,53	<0,001	0,90	0,64	1,25	0,52
10 Dysarthria	0,46	0,37	0,59	<0,001	0,82	0,61	1,11	0,19
11 Neglect	0,39	0,28	0,54	<0,001	0,87	0,57	1,32	0,51

Supplemetary table 1. Logistic regression; dichotomized NIHSS items sub-score stratified for stroke subtypes, and outcome mRS≤1

	Ischemic stroke				Hemorrhage			
	Crude		Adjusted§		Crude		Adjusted†	
NIHSS	OR	95% CI	OR	95% CI	OR	95% CI	OR	95% CI
1a Consciousness	0.29	0.17-0.51	0.70	0.37-1.30	0.21	0.05-0.97	0.83	0.13-5.30
1b Orientation (0 ref.)	0.34	0.25-0.45	0.83	0.58-1.21	0.52	0.23-1.18	1.42	0.48-4.18
1c Commands	0.31	0.19-0.50	0.90	0.49-1.66	0.21	0.05-0.99	0.31	0.03-2.76
2 Best Gaze	0.29	0.19-0.42	*0.57*	*0.34-0.94*	0.77	0.29-2.06	2.36	0.59-9.41
3 Visual field	*0.34*	*0.25-0.47*	*0.54*	*0.36-0.82*	0.97	0.44-2.16	1.54	0.55-4.30
4 Facial Palsy	0.55	0.43-0.69	0.89	0.66-1.22	0.59	0.27-1.29	1.81	0.59-5.57
5 Motor Arm								
Right	0.33	0.21-050	0.60	0.35-1.02	*0.32*	*0.11-0.90*	0.56	0.14-2.17
Left	0.38	0.27-0.53	0.73	0.48-1.11	0.53	0.22-1.31	1.19	0.37-3.85
6 Motor Leg								
Right	*0.27*	*0.18-0.41*	*0.58*	*0.35-0.97*	0.37	0.13-1.08	0.63	0.16-2.45
Left	0.38	0.27-0.54	0.69	0.45-1.05	0.50	0.21-1.17	0.95	0.30-3.08
7 Ataxia	0.49	0.36-0.68	0.71	0.49-1.03	0.94	0.35-2.59	1.01	0.30-3.38
8 Sensory	0.55	0.42-0.72	0.85	0.61-1.20	0.28	0.12-061	0.42	0.16-1.12
9 Best Language	0.43	0.33-0.57	0.95	0.66-1.36	0.30	0.13-0.70	0.58	0.21-1.60
10 Dysarthria	0.50	0.39-0.63	0.86	0.63-1.18	0.29	0.13-064	0.46	0.17-1.24
11 Neglect	0.36	0.25-0.52	0.75	0.47-1.19	0.69	0.29-1.64	1.83	0.57-5.83
Motor arm symptoms	0.31	0.24-0.42	*0.56*	*0.37-0.84*	0.30	0.14-0.64	0.60	0.19-1.91
Motor leg symptoms	0.30	0.23-0.40	*0.53*	*0.36-0.79*	0.33	0.15-0.72	0.60	0.18-1.94
Right motor symptoms	0.30	0.21-0.44	0.63	0.40-1.00	0.39	0.16-0.99	0.74	0.23-2.38
Left motor symptoms	0.40	0.29-0.54	0.69	0.48-1.00	0.51	0.22-1.16	0.90	0.29-2.76
Motor symptoms in at least one limb	0.32	0.25-0.41	0.55	0.38-0.79	0.32	0.15-0.68	0.63	0.20-2.05

§Adjusted for age, gender, premorbid independency, stroke severity and reperfusion therapy
† Adjusted for age, gender, premorbid independency and stroke severity
NIHSS=National Institutes of Health Stroke Scale, OR=odds ratio, CI=confidence interval
Each NIHSS item component on admission was dichotomized into; no symptoms (score 0) or symptoms (score of 1 or more).
Significant values in Italic.

For peer review only

Supplemental table 2: Characteristics of patients not included

**Total**	**2269**
**Age**	
**Mean (SD)**	75 (14)*
**Median (IQR)**	77 (85-67)
**Sex (female) (%)**	1150 (51)*
**Stroke type (%)***	
**Ischemic**	1955 (86)
**Hemorrhagic**	350 (13)
**Reperfusion therapy (%)**	242 (16.5)
**NIHSS**	
**Mean (SD)**	7 (8)*
**Median (IQR)**	2 (8-0)
**Stroke severity (%)***	
**Mild (NIHSS≤3)**	1107 (49)
**Mild to moderate**	777 (34)
**Severe (NIHSS>15)**	380 (17)
**mRS 3 months (n = 293)**	
**Median (IQR)**	2 (3-1)
**Excellent outcome (mRS≤1)**	43 (15)*
**Discharged to (%)**	
**Home**	1252 (55)*
**Premorbid independency* (%)**	1169 (79.5)
**NIHSS sub score (%)**	
**1a Consciousness (n = 1382)**	125 (9)*
**1b Orientation (n = 1366)**	410 (30)*
**1c Commands (n = 1357)**	170 (13)*
**2 Best Gaze (n = 1329)**	240 (18)*
**3 Visual field (n = 1304)**	280 (21)*
**4 Facial palsy (n = 1332)**	454 (30.9)*
**5 Motor arm**	
**Right (n = 1354)**	213 (16)*
**Left (n = 1356)**	220 (16)
**6 Motor leg**	
**Right (n = 1348)**	222 (16)*
**Left (n = 1350)**	229 (17)*
**7 Ataxia (n = 1313)**	232 (18)
**8 Sensory (n = 1321)**	386 (29)*
**9 Best Language (n = 1317)**	422 (32)*
**10 Dysarthria (n = 1310)**	464 (35)*
**11 Neglect (n = 1256)**	194 (15)
**Cardiovascular risk/comorbidity (%)**	
**Atrial fibrillation**	450 (20)*
**Diabetes**	408 (18)
**Smoking**	291 (13)*
**On statins**	504 (22)
**Hypertension**	1210 (53)*

*signficant different from included cases. SD standard deviation, IQR interquartile range, NIHSS=National Institutes of Health Stroke Scale, mRS=modified Rankin scale

For peer review only

For peer review only - http://bmjopen.bmj.com/site/about/guidelines.xhtml

STROBE Statement—Checklist of items that should be included in reports of ***cohort studies***

	Item No	Recommendation	Page No
**Title and abstract**	1	(*a*) Indicate the study's design with a commonly used term in the title or the abstract	2
		(*b*) Provide in the abstract an informative and balanced summary of what was done and what was found	2
**Introduction**			
Background/rationale	2	Explain the scientific background and rationale for the investigation being reported	3
Objectives	3	State specific objectives, including any prespecified hypotheses	3
**Methods**			
Study design	4	Present key elements of study design early in the paper	3-4
Setting	5	Describe the setting, locations, and relevant dates, including periods of recruitment, exposure, follow-up, and data collection	3-4
Participants	6	(*a*) Give the eligibility criteria, and the sources and methods of selection of participants. Describe methods of follow-up	4
		(*b*) For matched studies, give matching criteria and number of exposed and unexposed	
Variables	7	Clearly define all outcomes, exposures, predictors, potential confounders, and effect modifiers. Give diagnostic criteria, if applicable	4
Data sources/ measurement	8*	For each variable of interest, give sources of data and details of methods of assessment (measurement). Describe comparability of assessment methods if there is more than one group	4
Bias	9	Describe any efforts to address potential sources of bias	
Study size	10	Explain how the study size was arrived at	4
Quantitative variables	11	Explain how quantitative variables were handled in the analyses. If applicable, describe which groupings were chosen and why	4
Statistical methods	12	(*a*) Describe all statistical methods, including those used to control for confounding	4
		(*b*) Describe any methods used to examine subgroups and interactions	
		(*c*) Explain how missing data were addressed	
		(*d*) If applicable, explain how loss to follow-up was addressed	
		(*e*) Describe any sensitivity analyses	
**Results**			
Participants	13*	(a) Report numbers of individuals at each stage of study—eg numbers potentially eligible, examined for eligibility, confirmed eligible, included in the study, completing follow-up, and analysed	5 Flow chart
		(b) Give reasons for non-participation at each stage	
		(c) Consider use of a flow diagram	
Descriptive data	14*	(a) Give characteristics of study participants (eg demographic, clinical, social) and information on exposures and potential confounders	5 Table1 Suppl.
		(b) Indicate number of participants with missing data for each variable of interest	
		(c) Summarise follow-up time (eg, average and total amount)	
Outcome data	15*	Report numbers of outcome events or summary measures over time	

For peer review only - http://bmjopen.bmj.com/site/about/guidelines.xhtml

Main results	16	(*a*) Give unadjusted estimates and, if applicable, confounder-adjusted estimates and their precision (eg, 95% confidence interval). Make clear which confounders were adjusted for and why they were included<br>(*b*) Report category boundaries when continuous variables were categorized<br>(*c*) If relevant, consider translating estimates of relative risk into absolute risk for a meaningful time period	5
Other analyses	17	Report other analyses done—eg analyses of subgroups and interactions, and sensitivity analyses	5<br>Suppl.
**Discussion**			
Key results	18	Summarise key results with reference to study objectives	5
Limitations	19	Discuss limitations of the study, taking into account sources of potential bias or imprecision. Discuss both direction and magnitude of any potential bias	6
Interpretation	20	Give a cautious overall interpretation of results considering objectives, limitations, multiplicity of analyses, results from similar studies, and other relevant evidence	7
Generalisability	21	Discuss the generalisability (external validity) of the study results	6
**Other information**			
Funding	22	Give the source of funding and the role of the funders for the present study and, if applicable, for the original study on which the present article is based	7

*Give information separately for exposed and unexposed groups.

Note: An Explanation and Elaboration article discusses each checklist item and gives methodological background and published examples of transparent reporting. The STROBE checklist is best used in conjunction with this article (freely available on the Web sites of PLoS Medicine at http://www.plosmedicine.org/, Annals of Internal Medicine at http://www.annals.org/, and Epidemiology at http://www.epidem.com/). Information on the STROBE Initiative is available at http://www.strobe-statement.org.

For peer review only

BMJ Open

The prognostic value of acute National Institutes of Health Stroke Scale Items on disability: A Registry Study of first-ever-stroke in the western part of Sweden

Journal:	*BMJ Open*
Manuscript ID	bmjopen-2023-080007.R2
Article Type:	Original research
Date Submitted by the Author:	23-Nov-2023
Complete List of Authors:	Hagberg, Guri; Oslo universitetssykehus Ullevål, Neurological Department ; University of Gothenburg Institute of Neuroscience and Physiology, Section for Clinical Neuroscience and Rehabilitation<br>Ihle-Hansen, Haakon; Vestre Viken HF, Department of medical research; University of Oslo, Institute of Clinical Medicine<br>Abzhandadze, Tamar; University of Gothenburg, Department of Clinical Neuroscience, Rehabilitation Medicine; Sahlgrenska University Hospital Department of Occupational Therapy and Physiotherapy<br>Reinholdsson, Malin; University of Gothenburg Institute of Neuroscience and Physiology, Rehabilitation medicine; Sahlgrenska University Hospital Department of Occupational Therapy and Physiotherapy,<br>Hansen, Hege; Vestre Viken HF, Department of Medicine; Oslo University Hospital, Department of Neurology<br>Sunnerhagen, Katharina; University of Gothenburg Institute of Neuroscience and Physiology, Institute of Neuroscience and Physiology
**Primary Subject Heading**:	Neurology
Secondary Subject Heading:	Emergency medicine, Medical management
Keywords:	Stroke < NEUROLOGY, Prognosis, Stroke medicine < INTERNAL MEDICINE, REGISTRIES

SCHOLARONE™
Manuscripts

I, the Submitting Author has the right to grant and does grant on behalf of all authors of the Work (as defined in the below author licence), an exclusive licence and/or a non-exclusive licence for contributions from authors who are: i) UK Crown employees; ii) where BMJ has agreed a CC-BY licence shall apply, and/or iii) in accordance with the terms applicable for US Federal Government officers or employees acting as part of their official duties; on a worldwide, perpetual, irrevocable, royalty-free basis to BMJ Publishing Group Ltd ("BMJ") its licensees and where the relevant Journal is co-owned by BMJ to the co-owners of the Journal, to publish the Work in this journal and any other BMJ products and to exploit all rights, as set out in our licence.

The Submitting Author accepts and understands that any supply made under these terms is made by BMJ to the Submitting Author unless you are acting as an employee on behalf of your employer or a postgraduate student of an affiliated institution which is paying any applicable article publishing charge ("APC") for Open Access articles. Where the Submitting Author wishes to make the Work available on an Open Access basis (and intends to pay the relevant APC), the terms of reuse of such Open Access shall be governed by a Creative Commons licence – details of these licences and which Creative Commons licence will apply to this Work are set out in our licence referred to above.

Other than as permitted in any relevant BMJ Author's Self Archiving Policies, I confirm this Work has not been accepted for publication elsewhere, is not being considered for publication elsewhere and does not duplicate material already published. I confirm all authors consent to publication of this Work and authorise the granting of this licence.

For peer review only

The prognostic value of acute National Institutes of Health Stroke Scale Items on disability: A Registry Study of first-ever-stroke in the western part of Sweden

MD PhD Guri Hagberg[1,3] MD PhD Haakon Ihle-Hansen[2], OT PhD Tamar Abzhandadze[3,4], PT MSc Malin Reinholdsson[3,4], MD PhD Hege Ihle-Hansen[1,2], Prof Katharina S. Sunnerhagen[3,5]

[1]Oslo Stroke Unit, Department of Neurology, Oslo University Hospital, Ullevål, Norway.

[2]Bærum Hospital- Vestre Viken Hospital Trust, Department of Medicine, Drammen, Norway.

[3]Institute of Neuroscience and Physiology, Section for Clinical Neuroscience and Rehabilitation, Gothenburg University, Gothenburg, Sweden

[4]Department of Occupational Therapy and Physiotherapy, Sahlgrenska University Hospital, Gothenburg, Sweden

[5]Neurocare, Sahlgrenska University Hospital, Gothenburg, Sweden.

Corresponding author: Guri Hagberg, Institute of Neuroscience and Physiology, Section for Clinical Neuroscience and Rehabilitation, Gothenburg University, Gothenburg, Sweden.

Mail: guri.hagberg@gmail.com

Running title: The prognostic value of NIHSS sub-items

Keywords: Stroke, NIHSS, mRS, prognosis

Word count: 2602

For peer review only

Abstract

Objectives We aimed to study how the individual items of the National Institutes of Health Stroke Scale (NIHSS) at admission predict functional independence three months post-stroke in patients with first-ever stroke.

Setting This registry-based study used data from two Swedish stroke registers (Riksstroke, the mandatory national quality register for stroke care in Sweden and Väststroke, a local quality stroke register in Gothenburg).

Participants This study included patients with first-ever acute stroke admitted from November 2014 to August 2018, with available NIHSS at admission and modified Rankin Scale (mRS) at three months follow-up.

Primary outcome The primary outcome variable was mRS ≤1 (defined as an excellent outcome) at three months follow-up.

Results We included 1471 patients, mean age was 72 (± 14.5) years, 48 % were female, and 66 % had mild strokes (NIHSS ≤ 3). In adjusted binary logistic regression analysis, the NIHSS items impaired right motor arm and leg, and impairment in visual field, reduced the odds of an excellent outcome at three months ((OR 0.60 (95% CI: 0.37-0.98), OR 0.60 (95% CI: 0.37-0.97), and OR 0.65 (95% CI: 0.45-0.94)). When exploring the effect size of associations between NIHSS items and mRS ≤1p, orientation, language, and right leg motor had the largest yet small association.

Conclusions Stroke patients with scores on the NIHSS items right motor symptoms or visual field at admission are less likely to have excellent outcome at three months. Clinicians should consider the NIHSS items affected, not only the total NIHSS score, both in treatment guidance and prognostics.

Key words: Stroke, NIHSS, prognosis, mRS

Strengths and limitations of this study

- This study is based on registry data of high quality and high coverage, reflecting real-life clinical setting, potentially minimizing the risk of selection bias.
- Registry data have limitations with missing data that might limit the generalizability.
- To our knowledge, this study in the first to examine the prognostic merit of NIHSS individual items regardless of stroke severity on post-stroke functioning.

For peer review only - http://bmjopen.bmj.com/site/about/guidelines.xhtml

Introduction

The National Institutes of Health Stroke Scale (NIHSS) includes 15 individual items measuring motor and sensory function, vision, level of consciousness, attention, neglect, language, and speech production (1, 2). The NIHSS is the most widespread clinical scale used in patients presenting with acute stroke, and a high total NIHSS score is associated with poor outcomes in both hemorrhagic and ischemic strokes (3-5).

The total NIHSS score has several limitations as a predictor of later stroke outcomes. The 15 items are weighted similarly, scoring 0-42, but left brain strokes generally scores 4 points higher on the NIHSS than right brain strokes of larger volume (6). Total NIHSS scores at admission are inferior to 24 h and discharge scores in predicting post-stroke functional outcomes, due to spontaneous improvement and the effect of acute interventions like thrombolysis or thrombectomy (7, 8). In strokes affecting posterior circulation, a low NIHSS might not predict a good functional outcome as the NIHSS is biased towards anterior circulation strokes (9, 10). In addition, in minor stroke, defined as NIHSS ≤ 3p (11), up to one-third of the patients are disabled or dead after three months (12, 13).

As the stroke field evolves, physicians tend to treat more small strokes with reperfusion therapy, although the published papers supporting this are lacking (14). Current American Heart Association/American Stroke Association guidelines indicate uncertainty regarding the use of intravenous thrombolysis (IVT) in patients with low NIHSS scores and non-disabling deficits (15), and recent European guidelines suggest against IVT in acute minor non-disabling stroke (16). Disabling strokes is not clearly defined in clinical practice, and excluding patients from acute treatment because their symptoms are considered too mild by the treating physician, might lead to unnecessary disability and deaths (17, 18). A suggested definition is that a disabling deficit would prevent the patient from performing basic activities of daily living or returning to work (19), both clearly dependent on the patients premorbid function and age.

In light of the widespread clinical use of NIHSS in the acute phase defining stroke severity and the total NIHSS scores limitations, knowing how the individual NIHSS items relate to functional outcomes after three months is essential in both treatment guidance and prognostics. Since few stroke registers report the full NIHSS with sub-items, the evidence on the prognostic merit of NIHSS individual items regardless of stroke severity on post-stroke functioning is lacking. We aimed to study how the individual items of NIHSS at admission predicts functional independence, modified Rankin Scale (mRS) ≤1, three months post-stroke in an unselected stroke population.

Methods

Study design

This register-based study used data from two Swedish stroke registers (Riksstroke, the mandatory national quality register for stroke care in Sweden (20) and Väststroke, a local quality stroke register in Gothenburg, collecting data from 2014 to 2019). The two registers provide different information about the same patients. The datasets were merged by a

For peer review only - http://bmjopen.bmj.com/site/about/guidelines.xhtml

statistician at Riksstroke through personal identification numbers into one pseudonymized database.

Study sample

This study includes all the patients ≥ 18 years admitted with a first-ever acute stroke (ischemic stroke (I63) or intracerebral hemorrhage (I61) according to the International Classification of Diseases (ICD-10), to the Sahlgrenska University Hospital from November 2014 to August 2018, with available NIHSS items scores at admission and mRS at three months follow-up. Further, permanent institution residents were not invited to the follow-up, and together with patients with mRS = 6 (dead) excluded from the study. The Sahlgrenska University Hospital consists of three sites, each with a comprehensive stroke unit, and provides emergency and basic care for 800,000 inhabitants in Gothenburg as well as specialized care for 1.8 million inhabitants in the west of Sweden.

Procedure and study variables

Väststroke contains information on the NIHSS at admission, assessed by physicians. In cases with missing NIHSS in the Väststroke register, assessments were retrieved from medical records when possible (21). In Sweden, stroke patients are invited to a three-month outpatient follow-up visit for a medical review and assessment of functional status (mRS). The data on mRS were collected from the charts or by telephone from the nurses in a structured way (22). All variables in this study with the exception of mRS at 3 months were collected at the index stay. In Riksstroke the data were recorded by trained nurses working at the stroke units. Data retrieved from Riksstroke includes variables like sex, age, vascular risk factors, reperfusion treatment, and discharge destination. The explanatory variables where NIHSS items, and each NIHSS item component on admission was dichotomized into; no symptom (score 0) or symptom(s) (score of 1 or more). The outcome variable was mRS, and an excellent outcome was defined as mRS ≤1.

Statistics

Descriptive demographic and clinical measures are given as mean ± SD, median and interquartile range (IQR) or as numbers and percentages as appropriate. Categorical variables were compared with chi-square ($\chi 2$) and Mann–Whitney U-test for continuous variables. Participants were divided into two groups based on mRS levels (mRS ≤ 1 indicating an excellent outcome or mRS > 1, indicating from slight to severe disability). In order to identify NIHSS items as predictors of an excellent outcome, logistic regression models were fitted and adjusted for the predefined variables such as age, sex, premorbid independency, stroke subtype, and admission stroke severity (according to NIHSS; minor 0-3, mild to moderate 4-15; severe ≥16). As an exploratory analysis, a second model stratified for the stroke subtypes, ischemic stroke or hemorrhage, and adjusted for age, sex, premorbid independency, stroke severity, and reperfusion therapy (in case of ischemic stroke), were built (Supplementary table 1). The results are given in odds ratios (OR) with a 95% confidence interval (CI). The effect size of the association between the excellent outcome and NIHSS items was investigated using phi coefficient; 0.1-0.29 (small effect), 0.30-0.49 (medium effect), and 0.5-1.0 (large effect) (23). All statistical tests were two sided with alpha 5 %. The analyses were performed using IBM SPSS Statistics 28 software.

For peer review only - http://bmjopen.bmj.com/site/about/guidelines.xhtml

Results

Out of 3740 patients admitted with acute stroke, 1471 (39 %) patients had available data on NIHSS items at admission and mRS at three months follow-up; see the flow chart in Figure 1. Among the 2269 not included patients, 374 (16.5 %) died during the hospital stay, and in general, they were older, with a mean age of 75 ± 13.5 years, and had higher mean NIHSS (6.9 ± 8) (Supplemental Table 2).

[insert figure 1]

Of 1471 included patients, 48 % were female, the mean age was 72 ± 14.5 years, and with mean NIHSS 3.8 ± 5.1 at stroke admission. Median (IQR) mRS at 3 months was 2 (2). Most (91 %) of the strokes were ischemic, and 66 % were considered mild with NIHSS ≤ 3. Facial palsy (in 31 % of the stroke cases), followed by dysarthria (30 %), was the most common scored NIHSS item. There were significantly higher proportion of patients with left side than right side motor symptoms ($p<0.001$). Patient characteristics and NIHSS items sub-scores are presented in Table 1. The distribution of mRS at three months based on stroke severity at admission is shown in Figure 2, where 26 % of patients with mild strokes had mRS ≤ 1 at three months.

[insert figure 2]

In binary logistic crude regression models, scores in any NIHSS sub-items significantly reduced the odds of an excellent outcome (mRS ≤ 1) at three months. After adjustments for the other independent variables (age, sex, premorbid independency, stroke subtype, and stroke severity), scores on the right arm and leg motor symptoms and visual fields reduced the odds of an excellent outcome (mRS ≤ 1) at three months, OR 0.60 (95% CI: 0.37-0.98), OR 0.60 (95% CI: 0.37-0.97), and OR 0.65 (95% CI: 0.45-0.94), respectively (Figure 3). When stratifying for ischemic or hemorrhagic stroke, the results were comparable for ischemic stroke to those for the entire cohort, but no NIHSS item or side predicted outcome in hemorrhagic stroke (Supplementary table 1).

[insert figure 3]

When exploring the effect size of association between dichotomized NIHSS items and excellent outcome three months after stroke (mRS ≤ 1), orientation, language, and right leg motor had the largest, jet small, association (0.202, 0.183, and 0.182, $p < 0.001$), respectively (Table 2).

Discussion

In this cross-sectional retrospective and register-based study, patients with first-ever stroke and scores on the NIHSS items right motor symptoms or visual field at admission were less likely to have an excellent outcome at three months.

A possible explanation for the importance of the NIHSS right motor items for prognosis is the weighting of motor symptoms in the mRS and affection of the dominant hemisphere. The mRS attempts to measure functional independence, incorporating the Word Health Organizations components of body function, activity, and participation, but domains such as cognition or language are not directly measured (24, 25). Abnormal motor items on the NIHSS in minor ischemic strokes (NIHSS <5) has been linked to poor outcome in two other

For peer review only - http://bmjopen.bmj.com/site/about/guidelines.xhtml

studies, but none of them looked at the side of lesions (26, 27). When assessing stroke outcomes by the side of involvement, the evidence is inconclusive, although one might think that affecting language and dominant hand function would negatively influence the outcome (28, 29). Several scale items require intact language function, and studies have shown that patients with left-sided stroke and total anterior circulation syndromes score 4 points higher on the NIHSS than right-sided stroke patients despite smaller stroke volumes on imaging (6, 30). The weighting of NIHSS items associated with left-side lesions makes the NIHSS less suitable for predicting outcomes for right-side strokes (31). Our study, looking at dichotomized sub-items, supports the importance of dominant hemisphere affection.

The NIHSS item visual field reduces the odds of an excellent outcome in our study. Several studies have shown that visual field defects impact functional ability, quality of life, ability to participate in rehabilitation, and poor long-term recovery (32-37). Visual field defects could lead to loss of driving license, automatically giving mRS > 1, and linked to the risk of social isolation, depression and risk of requiring placement in long-term care (38). The most common type of visual field loss is homonymous hemianopia, most common in strokes affecting posterior circulation, supplying optic radiations, occipital cortex and other brain areas involved in visual field (39). NIHSS is generally less sensitive to strokes affecting posterior circulation, commonly causing vertigo and impaired balance (40), and a low NIHSS does not reflect stroke severity (9). In our cohort, 17.5 % had visual field defects, fewer than in many other studies (39, 41). The screening nature of vision testing in the acute setting and many patients being unaware of their loss might explain this (42). Our finding underpins the importance of visual field defects and testing in patients with otherwise low total NIHSS.

Our results could suggest that in patients with right motor symptoms or visual field defects, IVT should not be withheld due to low NIHSS alone, in line with previous work (13). However, a recently published randomized clinical trial found that IVT was non-inferior to dual anti-platelet therapy regarding an excellent functional outcome at 90 days in minor non-disabling acute ischemic stroke. They do not report on NIHSS items but included patients with NIHSS ≤ 5, with less than or equal to 1 point on the sub-items, including limb weakness or vision (14). Both guidelines and study protocols describe minor or isolated non-disabling symptoms as reasons to withhold IVT, but no clear description exists. Suggested deficits are ataxia alone, sensory loss alone, dysarthria or mild aphasia alone, minimal weakness, isolated facial droop, or mild cortical hand (16, 19, 43). As in our study, two other studies have shown that motor symptoms predict poor outcome, but instead of vision, they report ataxia as a predictor (9, 26). However, this is not supported in our data.

In general, and in line with previous studies, the individual NIHSS items explained only a small portion of the variance in outcome (26, 44, 45). Long-term follow-up with sensitive outcome measures is needed to make good prediction models, especially in minor strokes.

The strength of this register-based study is an unselected stroke cohort, with a complete NIHSS score including sub-items and mRS at three months, collected in a real-life clinical setting, not excluding patients due to strict inclusion criteria. The overall coverage ratio in the Riksstroke register is 90 % (46), and taken together with the other factors mentioned above, the generalizability of our findings to countries with similar stroke populations and healthcare systems seems likely.

A limitation in our study is missing data, where only 39 % had both available data on NIHSS items at admission and mRS at three months follow-up. However, as our drop-out analyses show that patients excluded had larger strokes and higher mortality, our conclusions can contribute to the decision-making process in the acute treatment of minor stroke were

For peer review only - http://bmjopen.bmj.com/site/about/guidelines.xhtml

selection to and effect of IVT are less established. Another limitation is that we do not know the time from symptoms onset to NIHSS assessment, and studies have shown that NIHSS at 24 hours is better in prognostics, as many symptoms resolve during the first 24 hours (7).

In conclusion, stroke patients with scores on the NIHSS items right motor symptoms or visual field at admission are less likely to have an excellent outcome at three months. Clinicians should consider the NIHSS items affected, not only the total NIHSS score, both in treatment guidance and prognostics.

Patient and public involvement

There were no patient or members of the public involved in the planning of this study.

Ethics

The study was approved by the Regional Ethical Review Board in Gothenburg, Sweden on May 4, 2016, registration number 346-16, with an amendment approved on September 14, 2018, registration number T807-18. According to the Data Inspection Board in Sweden data from quality registers are exempt from the general rule of patient consent (the Personal Data Act, Swedish law No. SFS 1998:204), since their purpose is to improve the quality of care, treatment, and rehabilitation which is of public interest. Furthermore, the patients were informed that their data were reported to the stroke registers and could be used for research. The patients may opt-out with withdrawal of their data at any time. The data were pseudonymized, thus patients cannot be identified.

Author Contributors

G. Hagberg: drafting of the manuscript. M. Reinholdsson, T. Abzhandadze: acquisition of data. G. Hagberg, M. Reinholdsson, T. Abzhandadze, H. Ihle-Hansen, H. Ihle-Hansen and K.S. Sunnerhagen contributed to study concept and design, analysis and interpretation of data and critical revision of the manuscript for intellectual content.

Data sharing statement

Data from registries are subject to the Personal Data Act (Swedish law No. SFS 1998:204). Data may be available to researchers upon request, after review of secrecy (contact the author ks.sunnerhagen@neuro.gu.se). According to the Swedish regulation (epn.se/en/start/regulations/), the permission to use data can only be according to application and approval from the ethical board.

Disclosure of interest

The authors declares that there is no conflict of interest regarding the publication of this paper.

Sources of funding

This work was supported by The Local Research and Development Board for Gothenburg and Södra Bohuslän, the Renée Eanders Foundation, the Swedish Stroke Association, the Swedish Heart-Lung Foundation, the Swedish Brain Foundation, Promobilia, and the Swedish Research Council VR 2017-00946. The Swedish state under the ALF agreement, an agreement between the Swedish government and county councils (ALFGBG-965653).

For peer review only
For peer review only - http://bmjopen.bmj.com/site/about/guidelines.xhtml

Acknowledgment

The authors thank the members of the Riksstroke Collaboration for data from the Swedish Stroke Register, Riksstroke (riksstroke.org), and the Väststroke Collaboration for data from the Väststroke Register. They also thank the participating patients and support staff for assistance with data acquisition.

References

1. Brott T, Adams HP, Jr., Olinger CP, Marler JR, Barsan WG, Biller J, et al. Measurements of acute cerebral infarction: a clinical examination scale. Stroke. 1989;20(7):864-70.
2. Goldstein LB, Bertels C, Davis JN. Interrater reliability of the NIH stroke scale. Archives of neurology. 1989;46(6):660-2.
3. Broderick JP, Lu M, Kothari R, Levine SR, Lyden PD, Haley EC, et al. Finding the most powerful measures of the effectiveness of tissue plasminogen activator in the NINDS tPA stroke trial. Stroke. 2000;31(10):2335-41.
4. Adams HP, Jr., Davis PH, Leira EC, Chang KC, Bendixen BH, Clarke WR, et al. Baseline NIH Stroke Scale score strongly predicts outcome after stroke: A report of the Trial of Org 10172 in Acute Stroke Treatment (TOAST). Neurology. 1999;53(1):126-31.
5. Finocchi C, Balestrino M, Malfatto L, Mancardi G, Serrati C, Gandolfo C. National Institutes of Health Stroke Scale in patients with primary intracerebral hemorrhage. Neurol Sci. 2018;39(10):1751-5.
6. Lyden P, Claesson L, Havstad S, Ashwood T, Lu M. Factor analysis of the National Institutes of Health Stroke Scale in patients with large strokes. Arch Neurol. 2004;61(11):1677-80.
7. Abdul-Rahim AH, Fulton RL, Sucharew H, Kleindorfer D, Khatri P, Broderick JP, et al. National Institutes of Health Stroke Scale Item Profiles as Predictor of Patient Outcome: External Validation on Safe Implementation of Thrombolysis in Stroke-Monitoring Study Data. Stroke. 2015;46(10):2779-85.
8. Reznik ME, Yaghi S, Jayaraman MV, McTaggart RA, Hemendinger M, Mac Grory BC, et al. Baseline NIH Stroke Scale is an inferior predictor of functional outcome in the era of acute stroke intervention. International journal of stroke : official journal of the International Stroke Society. 2018;13(8):806-10.
9. Sato S, Toyoda K, Uehara T, Toratani N, Yokota C, Moriwaki H, et al. Baseline NIH Stroke Scale Score predicting outcome in anterior and posterior circulation strokes. Neurology. 2008;70(24 Pt 2):2371-7.
10. Inoa V, Aron AW, Staff I, Fortunato G, Sansing LH. Lower NIH stroke scale scores are required to accurately predict a good prognosis in posterior circulation stroke. Cerebrovasc Dis. 2014;37(4):251-5.
11. Fischer U, Baumgartner A, Arnold M, Nedeltchev K, Gralla J, De Marchis GM, et al. What is a minor stroke? Stroke. 2010;41(4):661-6.
12. Khatri P, Conaway MR, Johnston KC. Ninety-day outcome rates of a prospective cohort of consecutive patients with mild ischemic stroke. Stroke. 2012;43(2):560-2.
13. Wendt M, Tütüncü S, Fiebach JB, Scheitz JF, Audebert HJ, Nolte CH. Preclusion of ischemic stroke patients from intravenous tissue plasminogen activator treatment for mild symptoms should not be based on low National Institutes of Health Stroke Scale Scores. J Stroke Cerebrovasc Dis. 2013;22(4):550-3.
14. Chen HS, Cui Y, Zhou ZH, Zhang H, Wang LX, Wang WZ, et al. Dual Antiplatelet Therapy vs Alteplase for Patients With Minor Nondisabling Acute Ischemic Stroke: The ARAMIS Randomized Clinical Trial. Jama. 2023;329(24):2135-44.
15. Powers WJ, Rabinstein AA, Ackerson T, Adeoye OM, Bambakidis NC, Becker K, et al. Guidelines for the Early Management of Patients With Acute Ischemic Stroke: 2019 Update to the

For peer review only - http://bmjopen.bmj.com/site/about/guidelines.xhtml

2018 Guidelines for the Early Management of Acute Ischemic Stroke: A Guideline for Healthcare Professionals From the American Heart Association/American Stroke Association. Stroke; a journal of cerebral circulation. 2019;50(12):e344-e418.
16. Berge E, Whiteley W, Audebert H, De Marchis GM, Fonseca AC, Padiglioni C, et al. European Stroke Organisation (ESO) guidelines on intravenous thrombolysis for acute ischaemic stroke. Eur Stroke J. 2021;6(1):I-lxii.
17. Barber PA, Zhang J, Demchuk AM, Hill MD, Buchan AM. Why are stroke patients excluded from TPA therapy? An analysis of patient eligibility. Neurology. 2001;56(8):1015-20.
18. Duan C, Xiong Y, Gu HQ, Wang S, Yang KX, Hao M, et al. Outcomes in minor stroke patients treated with intravenous thrombolysis. CNS Neurosci Ther. 2023;29(8):2308-17.
19. Khatri P, Kleindorfer DO, Devlin T, Sawyer RN, Jr., Starr M, Mejilla J, et al. Effect of Alteplase vs Aspirin on Functional Outcome for Patients With Acute Ischemic Stroke and Minor Nondisabling Neurologic Deficits: The PRISMS Randomized Clinical Trial. Jama. 2018;320(2):156-66.
20. Asplund K, Hulter Åsberg K, Appelros P, Bjarne D, Eriksson M, Johansson A, et al. The Riks-Stroke story: building a sustainable national register for quality assessment of stroke care. International journal of stroke : official journal of the International Stroke Society. 2011;6(2):99-108.
21. Kasner SE, Cucchiara BL, McGarvey ML, Luciano JM, Liebeskind DS, Chalela JA. Modified National Institutes of Health Stroke Scale can be estimated from medical records. Stroke; a journal of cerebral circulation. 2003;34(2):568-70.
22. Bruno A, Akinwuntan AE, Lin C, Close B, Davis K, Baute V, et al. Simplified modified rankin scale questionnaire: reproducibility over the telephone and validation with quality of life. Stroke. 2011;42(8):2276-9.
23. Cohen J. A power primer. Psychol Bull. 1992;112(1):155-9.
24. van Swieten JC, Koudstaal PJ, Visser MC, Schouten HJ, van Gijn J. Interobserver agreement for the assessment of handicap in stroke patients. Stroke. 1988;19(5):604-7.
25. Kasner SE. Clinical interpretation and use of stroke scales. The Lancet Neurology. 2006;5(7):603-12.
26. Yaghi S, Willey JZ, Andrews H, Boehme AK, Marshall RS, Boden-Albala B. The Itemized NIHSS Scores Are Associated With Discharge Disposition in Patients With Minor Stroke. Neurohospitalist. 2016;6(3):102-6.
27. Sato S, Uehara T, Ohara T, Suzuki R, Toyoda K, Minematsu K. Factors associated with unfavorable outcome in minor ischemic stroke. Neurology. 2014;83(2):174-81.
28. Di Legge S, Saposnik G, Nilanont Y, Hachinski V. Neglecting the difference: does right or left matter in stroke outcome after thrombolysis? Stroke. 2006;37(8):2066-9.
29. Fink JN, Frampton CM, Lyden P, Lees KR. Does hemispheric lateralization influence functional and cardiovascular outcomes after stroke?: an analysis of placebo-treated patients from prospective acute stroke trials. Stroke. 2008;39(12):3335-40.
30. Woo D, Broderick JP, Kothari RU, Lu M, Brott T, Lyden PD, et al. Does the National Institutes of Health Stroke Scale favor left hemisphere strokes? NINDS t-PA Stroke Study Group. Stroke. 1999;30(11):2355-9.
31. Yoo AJ, Romero J, Hakimelahi R, Nogueira RG, Rabinov JD, Pryor JC, et al. Predictors of functional outcome vary by the hemisphere of involvement in major ischemic stroke treated with intra-arterial therapy: a retrospective cohort study. BMC Neurol. 2010;10:25.
32. Jones SA, Shinton RA. Improving outcome in stroke patients with visual problems. Age Ageing. 2006;35(6):560-5.
33. Leff AP, Scott SK, Crewes H, Hodgson TL, Cowey A, Howard D, et al. Impaired reading in patients with right hemianopia. Ann Neurol. 2000;47(2):171-8.
34. Ramrattan RS, Wolfs RC, Panda-Jonas S, Jonas JB, Bakker D, Pols HA, et al. Prevalence and causes of visual field loss in the elderly and associations with impairment in daily functioning: the Rotterdam Study. Arch Ophthalmol. 2001;119(12):1788-94.
35. West CG, Gildengorin G, Haegerstrom-Portnoy G, Schneck ME, Lott L, Brabyn JA. Is vision function related to physical functional ability in older adults? J Am Geriatr Soc. 2002;50(1):136-45.

For peer review only - http://bmjopen.bmj.com/site/about/guidelines.xhtml

36. Sand KM, Wilhelmsen G, Naess H, Midelfart A, Thomassen L, Hoff JM. Vision problems in ischaemic stroke patients: effects on life quality and disability. Eur J Neurol. 2016;23 Suppl 1:1-7.
37. Johnson N, Nisar T, Criswell A, McCane D, Lee J, Chiu D, et al. Long-Term Disability Outcomes for Patients With Ischemic Stroke Presenting With Visual Deficits. J Neuroophthalmol. 2022;42(4):518-23.
38. Owsley C, McGwin G, Jr. Vision and driving. Vision Res. 2010;50(23):2348-61.
39. Rowe FJ, Wright D, Brand D, Jackson C, Harrison S, Maan T, et al. A prospective profile of visual field loss following stroke: prevalence, type, rehabilitation, and outcome. Biomed Res Int. 2013;2013:719096.
40. Libman RB, Kwiatkowski TG, Hansen MD, Clarke WR, Woolson RF, Adams HP. Differences between anterior and posterior circulation stroke in TOAST. Cerebrovasc Dis. 2001;11(4):311-6.
41. Gray CS, French JM, Bates D, Cartlidge NE, Venables GS, James OF. Recovery of visual fields in acute stroke: homonymous hemianopia associated with adverse prognosis. Age Ageing. 1989;18(6):419-21.
42. Falke P, Abela BM, Jr., Krakau CE, Lilja B, Lindgärde F, Maly P, et al. High frequency of asymptomatic visual field defects in subjects with transient ischaemic attacks or minor strokes. J Intern Med. 1991;229(6):521-5.
43. Powers WJ, Rabinstein AA, Ackerson T, Adeoye OM, Bambakidis NC, Becker K, et al. 2018 Guidelines for the Early Management of Patients With Acute Ischemic Stroke: A Guideline for Healthcare Professionals From the American Heart Association/American Stroke Association. Stroke; a journal of cerebral circulation. 2018;49(3):e46-e110.
44. Leira EC, Ludwig BR, Gurol ME, Torner JC, Adams HP, Jr. The types of neurological deficits might not justify withholding treatment in patients with low total National Institutes of Health Stroke Scale scores. Stroke. 2012;43(3):782-6.
45. Choi JC, Kim BJ, Han MK, Lee SJ, Kang K, Park JM, et al. Utility of Items of Baseline National Institutes of Health Stroke Scale as Predictors of Functional Outcomes at Three Months after Mild Ischemic Stroke. J Stroke Cerebrovasc Dis. 2017;26(6):1306-13.
46. Söderholm A, Stegmayr B, Glader EL, Asplund K. Validation of Hospital Performance Measures of Acute Stroke Care Quality. Riksstroke, the Swedish Stroke Register. Neuroepidemiology. 2016;46(4):229-34.

For peer review only

For peer review only - http://bmjopen.bmj.com/site/about/guidelines.xhtml

Table 1: Characteristics of the study sample

**Total**	**1471**
**Age**	
Mean (SD)	71.8 (14.5)
Median (IQR)	74 (82-63)
**Sex (female) (%)**	658 (47.7)
**Stroke type (%)**	
Ischemic	1335 (90.8)
Hemorrhagic	136 (9.2)
**Reperfusion therapy (%)**	242 (16.5)
**NIHSS**	
Mean (SD)	3.8 (5.1)
Median (IQR)	2 (5-0)
**Stroke severity (%)**	
Mild (NIHSS≤3)	965 (65.6)
Mild to moderate	429 (29.2)
Severe (NIHSS>15)	77 (5.2)
**mRS 3 months**	
Median (IQR)	2 (3-1)
Excellent outcome (mRS≤1)	703 (47.8)
**Discharged to (%)**	
Home	1045 (71.0)
Premorbid independency* (%)	1169 (79.5)
**NIHSS sub score (% of 1471)**	
1a Consciousness	90 (6.1)
1b Orientation	311 (21.1)
1c Commands	110 (7.5)
2 Best Gaze	169 (11.5)
3 Visual field	257 (17.5)
4 Facial palsy	454 (30.9)
5 Motor arm	
Right	147 (10.0)
Left	211 (14.3)
6 Motor leg	
Right	159 (10.8)
Left	213 (14.5)
7 Ataxia	206 (14.0)
8 Sensory	350 (23.8)
9 Best Language	340 (23.1)
10 Dysarthria	438 (29.8)
11 Neglect	183 (12.4)
**Cardiovascular risk/comorbidity (%)**	
Atrial fibrillation	310 (21.1)
Diabetes	246 (16.7)
Smoking	171 (11.6)
On statins	300 (20.4)
Hypertension	720 (48.9)

*140 missing, SD standard deviation, IQR interquartile range, NIHSS=National Institutes of Health Stroke Scale, mRS=modified Rankin scale

For peer review only

For peer review only - http://bmjopen.bmj.com/site/about/guidelines.xhtml

Table 2. Exploring the strength of relationship between dichotomized NIHSS items and excellent outcome (mRS≤1)

N (%)		Outcome according to mRS mRS>1 n (%)	mRS≤1 n (%)	χ2	Phi coefficient
1a Consciousness					
Absence		690 (90.7)	683 (97.3)		
Symptoms		71 (9.3)	19 (2.7)	<0.001	0.138
1b Orientation (0 ref.)					
Absence		538 (70.7)	611 (87.4)		
Symptoms		223 (29.3)	88 (12.6)	<0.001	0.204
1c Commands					
Absence		671 (88.8)	677 (96.4)		
Symptoms		85 (11.2)	25 (3.6)	<0.001	0.145
2 Best Gaze					
Absence		608 (82.8)	655 (93.8)		
Symptoms		126 (17.2)	43 (6.2)	<0.001	0.171
3 Visual field					
Absence		545 (75.2)	615 (88.9)		
Symptoms		180 (24.8)	77 (11.1)	<0.001	0.178
4 Facial palsy					
Absence		462 (62.3)	524 (75.1)		
Symptoms		280 (37.7)	174 (24.9)	<0.001	0.138
5 Motor arm					
Right					
	Absence	643 (85.3)	665 (94.9)		
	Symptoms	111 (14.7)	36 (5.1)	<0.001	0.159
Left					
	Absence	607 (80.2)	639 (91.3)		
	Symptoms	150 (19.8)	61 (8.7)	<0.001	0.158
6 Motor leg					
Right					
	Absence	619 (83.4)	664 (94.9)		
	Symptoms	123 (16.5)	36 (5.1)	<0.001	0.182
Left					
	Absence	595 (79.8)	637 (91.1)		
	Symptoms	151 (20.2)	62 (8.9)	<0.001	0.160
7 Ataxia					
Absence		590 (81.6)	619 (89.5)		
Symptoms		133 (18.4)	73 (10.5)	<0.001	0.111
8 Sensory					
Absence		509 (69.3)	556 (81.9)		
Symptoms		225 (30.7)	125 (18.1)	<0.001	0.146
9 Best Language					
Absence		500 (68.5)	582 (84.1)		
Symptoms		230 (31.5)	110 (15.9)	<0.001	0.183
10 Dysarthria					
Absence		448 (61.5)	539 (77.4)		
Symptoms		281 (38.5)	157 (22.6)	<0.001	0.173
11 Neglect					
Absence		561 (81.4)	625 (91.9)		
Symptoms		128 (18.6)	55 (8.1)	<0.001	0.154

Determining the effect size of the associations; Effect size 0.1-0.29 (small), 0.30-0.49 (medium) and 0.5-1.0 (large), mRS=modified Rankin scale. Each NIHSS item component on admission was dichotomized into; no symptoms (score 0) or symptoms (score of 1 or more).

For peer review only - http://bmjopen.bmj.com/site/about/guidelines.xhtml

Figure 1: Flow chart for study population

Figure 2: Stroke severity at baseline and mRS at 3 months

Percentage of patients with different mRS according to stroke severity (NIHSS; minor ≤ 3, mild to moderate 4-15; severe >15). NIHSS=National Institutes of Health Stroke Scale; mRS=modified Rankin scale.

Figure 3: Odds ratio for excellent outcome (mRS≤1) with NIHSS items sub-score in logistic regression (N = 1471)

*Unadjusted, †Adjusted for age, gender, premorbid independency, stroke subtype and stroke severity, OR=odds ratio, CI=confidence interval, NIHSS=National Institutes of Health Stroke Scale. Each NIHSS item component on admission was dichotomized into; no symptoms (score 0) or symptoms (score of 1 or more).

For peer review only

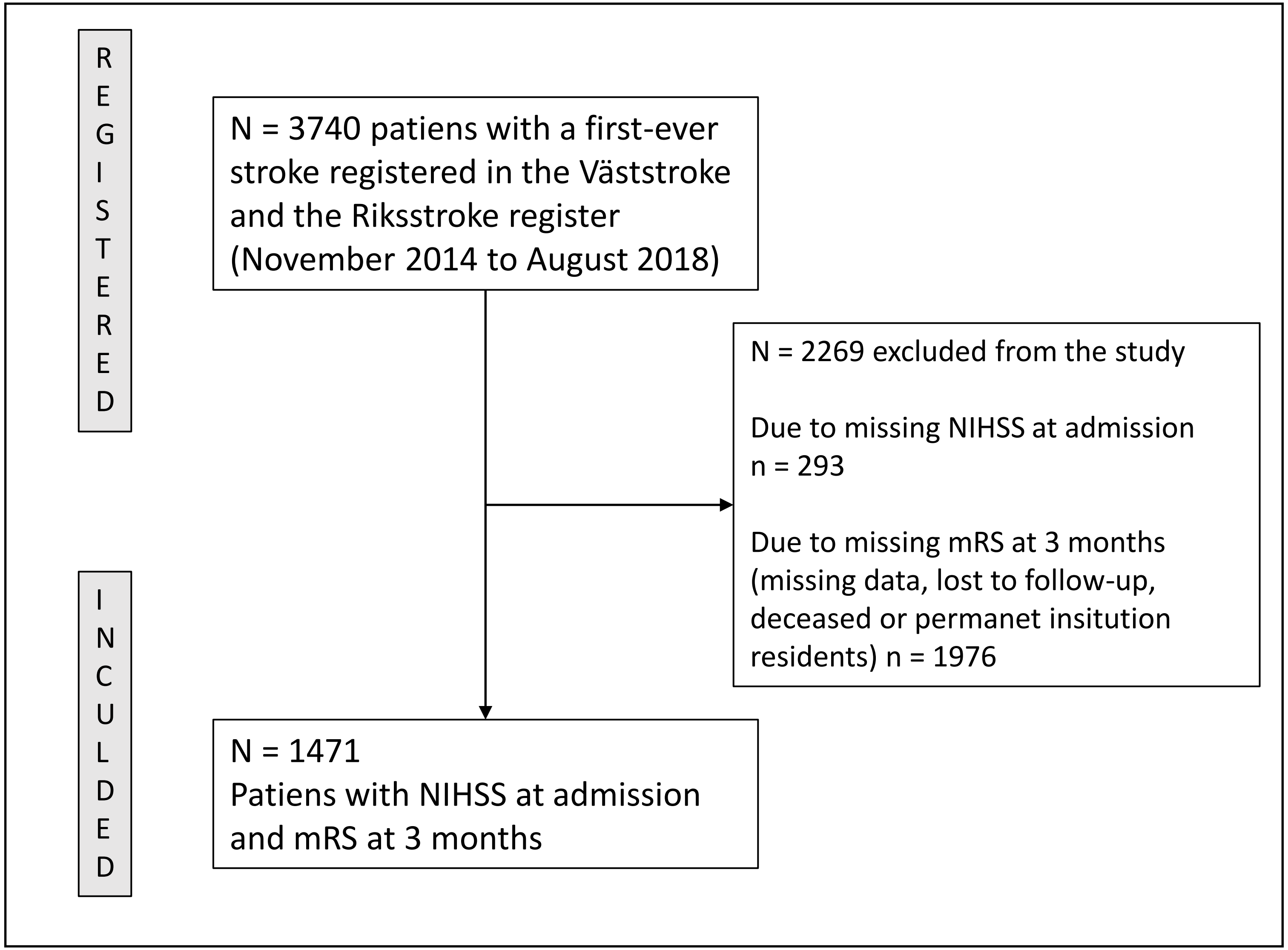
REGISTERED
N = 3740 patiens with a first-ever stroke registered in the Väststroke and the Riksstroke register (November 2014 to August 2018)
N = 2269 excluded from the study
Due to missing NIHSS at admission n = 293
Due to missing mRS at 3 months (missing data, lost to follow-up, deceased or permanet insitution residents) n = 1976
INCULDED
N = 1471
Patiens with NIHSS at admission and mRS at 3 months

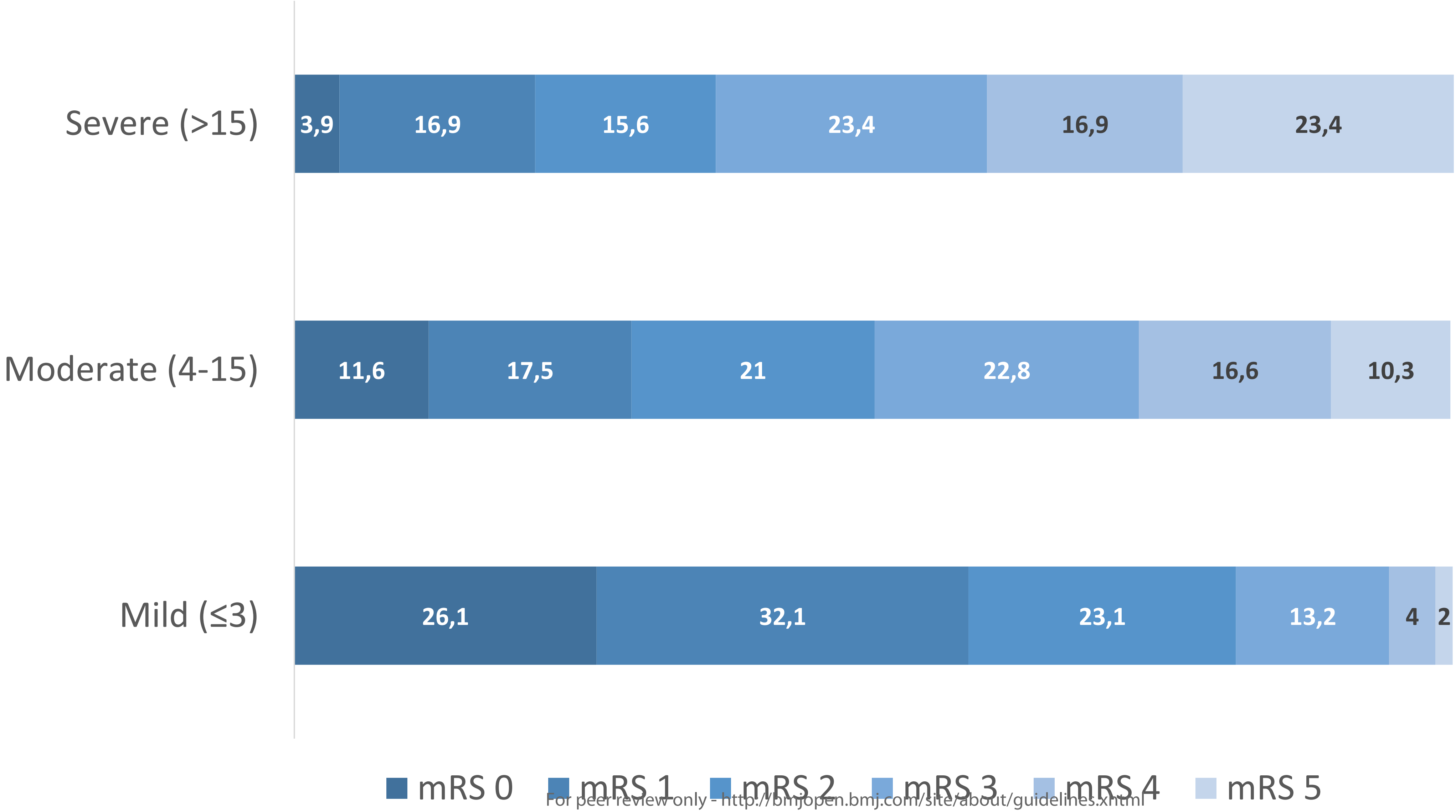

For peer review only - http://bmjopen.bmj.com/site/about/guidelines.xhtml

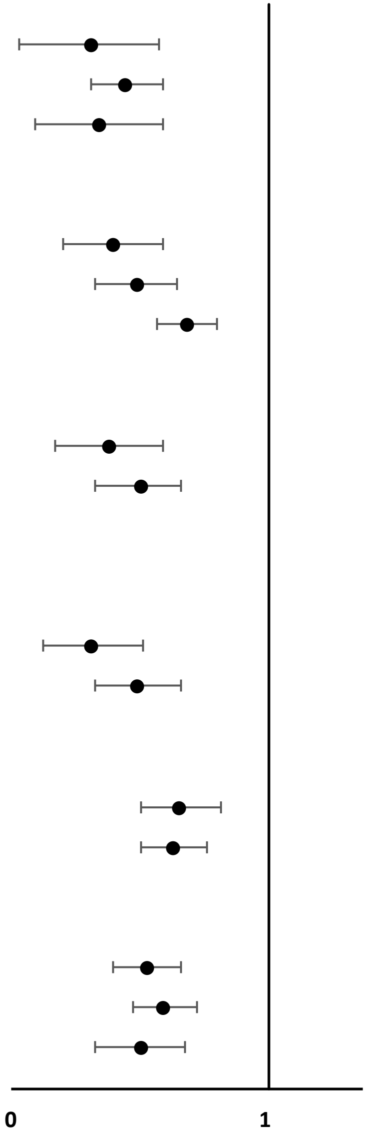

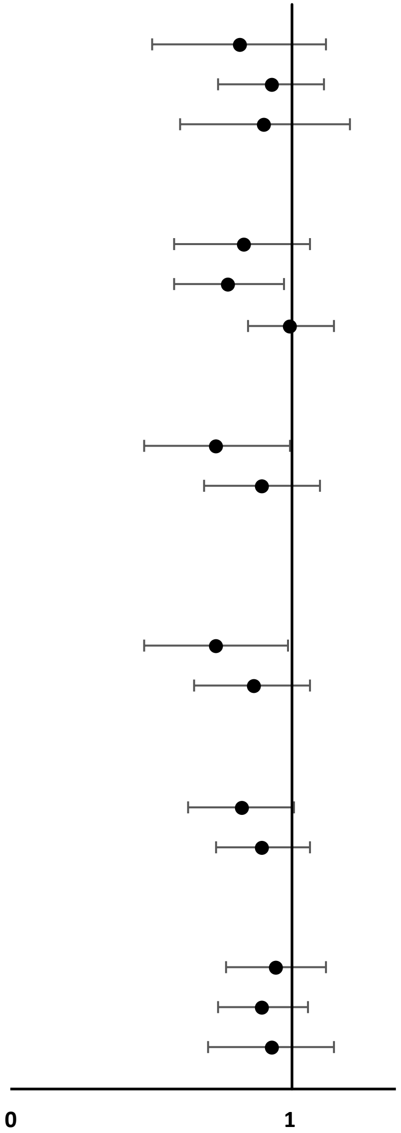

NIHSS sub items	mRS≤1* OR	95% CI		p-value	mRS≤1† OR	95% CI		p-value
1a Consciousness	0,27	0,16	0,45	<0,001	0,70	0,39	1,25	0,22
1b Orientation	0,35	0,27	0,46	<0,001	0,87	0,61	1,23	0,43
1c Commands	0,29	0,18	0,46	<0,001	0,83	0,47	1,48	0,53
2 Best Gaze	0,32	0,22	0,46	<0,001	0,72	0,45	1,13	0,15
3 Visual field	0,38	0,28	0,51	<0,001	0,65	0,45	0,94	0,02
4 Facial palsy	0,55	0,44	0,69	<0,001	0,99	0,74	1,32	0,93
5 Motor arm								
Right	0,31	0,21	0,46	<0,001	0,60	0,37	0,98	0,04
Left	0,39	0,28	0,53	<0,001	0,82	0,55	1,21	0,31
6 Motor leg								
Right	0,27	0,19	0,40	<0,001	0,60	0,37	0,97	0,04
Left	0,38	0,28	0,53	<0,001	0,77	0,52	1,13	0,18
7 Ataxia	0,52	0,39	0,71	<0,001	0,71	0,50	1,01	0,06
8 Sensory	0,50	0,39	0,64	<0,001	0,82	0,60	1,13	0,22
9 Best Language	0,41	0,32	0,53	<0,001	0,90	0,64	1,25	0,52
10 Dysarthria	0,46	0,37	0,59	<0,001	0,82	0,61	1,11	0,19
11 Neglect	0,39	0,28	0,54	<0,001	0,87	0,57	1,32	0,51

Supplemetary table 1. Logistic regression; dichotomized NIHSS items sub-score stratified for stroke subtypes, and outcome mRS≤1

	Ischemic stroke				Hemorrhage			
	Crude		Adjusted§		Crude		Adjusted†	
NIHSS	OR	95% CI	OR	95% CI	OR	95% CI	OR	95% CI
1a Consciousness	0.29	0.17-0.51	0.70	0.37-1.30	0.21	0.05-0.97	0.83	0.13-5.30
1b Orientation (0 ref.)	0.34	0.25-0.45	0.83	0.58-1.21	0.52	0.23-1.18	1.42	0.48-4.18
1c Commands	0.31	0.19-0.50	0.90	0.49-1.66	0.21	0.05-0.99	0.31	0.03-2.76
2 Best Gaze	0.29	0.19-0.42	*0.57*	*0.34-0.94*	0.77	0.29-2.06	2.36	0.59-9.41
3 Visual field	*0.34*	*0.25-0.47*	*0.54*	*0.36-0.82*	0.97	0.44-2.16	1.54	0.55-4.30
4 Facial Palsy	0.55	0.43-0.69	0.89	0.66-1.22	0.59	0.27-1.29	1.81	0.59-5.57
5 Motor Arm								
Right	0.33	0.21-050	0.60	0.35-1.02	*0.32*	*0.11-0.90*	0.56	0.14-2.17
Left	0.38	0.27-0.53	0.73	0.48-1.11	0.53	0.22-1.31	1.19	0.37-3.85
6 Motor Leg								
Right	*0.27*	*0.18-0.41*	*0.58*	*0.35-0.97*	0.37	0.13-1.08	0.63	0.16-2.45
Left	0.38	0.27-0.54	0.69	0.45-1.05	0.50	0.21-1.17	0.95	0.30-3.08
7 Ataxia	0.49	0.36-0.68	0.71	0.49-1.03	0.94	0.35-2.59	1.01	0.30-3.38
8 Sensory	0.55	0.42-0.72	0.85	0.61-1.20	0.28	0.12-061	0.42	0.16-1.12
9 Best Language	0.43	0.33-0.57	0.95	0.66-1.36	0.30	0.13-0.70	0.58	0.21-1.60
10 Dysarthria	0.50	0.39-0.63	0.86	0.63-1.18	0.29	0.13-064	0.46	0.17-1.24
11 Neglect	0.36	0.25-0.52	0.75	0.47-1.19	0.69	0.29-1.64	1.83	0.57-5.83
Motor arm symptoms	0.31	0.24-0.42	*0.56*	*0.37-0.84*	0.30	0.14-0.64	0.60	0.19-1.91
Motor leg symptoms	0.30	0.23-0.40	*0.53*	*0.36-0.79*	0.33	0.15-0.72	0.60	0.18-1.94
Right motor symptoms	0.30	0.21-0.44	0.63	0.40-1.00	0.39	0.16-0.99	0.74	0.23-2.38
Left motor symptoms	0.40	0.29-0.54	0.69	0.48-1.00	0.51	0.22-1.16	0.90	0.29-2.76
Motor symptoms in at least one limb	0.32	0.25-0.41	0.55	0.38-0.79	0.32	0.15-0.68	0.63	0.20-2.05

§Adjusted for age, gender, premorbid independency, stroke severity and reperfusion therapy
† Adjusted for age, gender, premorbid independency and stroke severity
NIHSS=National Institutes of Health Stroke Scale, OR=odds ratio, CI=confidence interval
Each NIHSS item component on admission was dichotomized into; no symptoms (score 0) or symptoms (score of 1 or more).
Significant values in Italic.

For peer review only

For peer review only - http://bmjopen.bmj.com/site/about/guidelines.xhtml

Supplemental table 2: Characteristics of patients not included

**Total**		**2269**
**Age**		
	**Mean (SD)**	75 (14)*
	**Median (IQR)**	77 (85-67)
**Sex (female) (%)**		1150 (51)*
**Stroke type (%)***		
	**Ischemic**	1955 (86)
	**Hemorrhagic**	350 (13)
**Reperfusion therapy (%)**		242 (16.5)
**NIHSS**		
	**Mean (SD)**	7 (8)*
	**Median (IQR)**	2 (8-0)
**Stroke severity (%)***		
	**Mild (NIHSS≤3)**	1107 (49)
	**Mild to moderate**	777 (34)
	**Severe (NIHSS>15)**	380 (17)
**mRS 3 months (n = 293)**		
	**Median (IQR)**	2 (3-1)
	**Excellent outcome (mRS≤1)**	43 (15)*
**Discharged to (%)**		
	**Home**	1252 (55)*
**Premorbid independency* (%)**		1169 (79.5)
**NIHSS sub score (%)**		
	**1a Consciousness (n = 1382)**	125 (9)*
	**1b Orientation (n = 1366)**	410 (30)*
	**1c Commands (n = 1357)**	170 (13)*
	**2 Best Gaze (n = 1329)**	240 (18)*
	**3 Visual field (n = 1304)**	280 (21)*
	**4 Facial palsy (n = 1332)**	454 (30.9)*
	**5 Motor arm**	
	**Right (n = 1354)**	213 (16)*
	**Left (n = 1356)**	220 (16)
	**6 Motor leg**	
	**Right (n = 1348)**	222 (16)*
	**Left (n = 1350)**	229 (17)*
	**7 Ataxia (n = 1313)**	232 (18)
	**8 Sensory (n = 1321)**	386 (29)*
	**9 Best Language (n = 1317)**	422 (32)*
	**10 Dysarthria (n = 1310)**	464 (35)*
	**11 Neglect (n = 1256)**	194 (15)
**Cardiovascular risk/comorbidity (%)**		
	**Atrial fibrillation**	450 (20)*
	**Diabetes**	408 (18)
	**Smoking**	291 (13)*
	**On statins**	504 (22)
	**Hypertension**	1210 (53)*

*signficant different from included cases. SD standard deviation, IQR interquartile range, NIHSS=National Institutes of Health Stroke Scale, mRS=modified Rankin scale

For peer review only

For peer review only - http://bmjopen.bmj.com/site/about/guidelines.xhtml

STROBE Statement—Checklist of items that should be included in reports of ***cohort studies***

	Item No	Recommendation	Page No
**Title and abstract**	1	(*a*) Indicate the study's design with a commonly used term in the title or the abstract	2
		(*b*) Provide in the abstract an informative and balanced summary of what was done and what was found	2
**Introduction**			
Background/rationale	2	Explain the scientific background and rationale for the investigation being reported	3
Objectives	3	State specific objectives, including any prespecified hypotheses	3
**Methods**			
Study design	4	Present key elements of study design early in the paper	3-4
Setting	5	Describe the setting, locations, and relevant dates, including periods of recruitment, exposure, follow-up, and data collection	3-4
Participants	6	(*a*) Give the eligibility criteria, and the sources and methods of selection of participants. Describe methods of follow-up	4
		(*b*) For matched studies, give matching criteria and number of exposed and unexposed	
Variables	7	Clearly define all outcomes, exposures, predictors, potential confounders, and effect modifiers. Give diagnostic criteria, if applicable	4
Data sources/ measurement	8*	For each variable of interest, give sources of data and details of methods of assessment (measurement). Describe comparability of assessment methods if there is more than one group	4
Bias	9	Describe any efforts to address potential sources of bias	
Study size	10	Explain how the study size was arrived at	4
Quantitative variables	11	Explain how quantitative variables were handled in the analyses. If applicable, describe which groupings were chosen and why	4
Statistical methods	12	(*a*) Describe all statistical methods, including those used to control for confounding	4
		(*b*) Describe any methods used to examine subgroups and interactions	
		(*c*) Explain how missing data were addressed	
		(*d*) If applicable, explain how loss to follow-up was addressed	
		(*e*) Describe any sensitivity analyses	
**Results**			
Participants	13*	(a) Report numbers of individuals at each stage of study—eg numbers potentially eligible, examined for eligibility, confirmed eligible, included in the study, completing follow-up, and analysed	5 Flow chart
		(b) Give reasons for non-participation at each stage	
		(c) Consider use of a flow diagram	
Descriptive data	14*	(a) Give characteristics of study participants (eg demographic, clinical, social) and information on exposures and potential confounders	5 Table1 Suppl.
		(b) Indicate number of participants with missing data for each variable of interest	
		(c) Summarise follow-up time (eg, average and total amount)	
Outcome data	15*	Report numbers of outcome events or summary measures over time	

For peer review only - http://bmjopen.bmj.com/site/about/guidelines.xhtml

Main results	16	(*a*) Give unadjusted estimates and, if applicable, confounder-adjusted estimates and their precision (eg, 95% confidence interval). Make clear which confounders were adjusted for and why they were included<br>(*b*) Report category boundaries when continuous variables were categorized<br>(*c*) If relevant, consider translating estimates of relative risk into absolute risk for a meaningful time period	5
Other analyses	17	Report other analyses done—eg analyses of subgroups and interactions, and sensitivity analyses	5<br>Suppl.
**Discussion**			
Key results	18	Summarise key results with reference to study objectives	5
Limitations	19	Discuss limitations of the study, taking into account sources of potential bias or imprecision. Discuss both direction and magnitude of any potential bias	6
Interpretation	20	Give a cautious overall interpretation of results considering objectives, limitations, multiplicity of analyses, results from similar studies, and other relevant evidence	7
Generalisability	21	Discuss the generalisability (external validity) of the study results	6
**Other information**			
Funding	22	Give the source of funding and the role of the funders for the present study and, if applicable, for the original study on which the present article is based	7

*Give information separately for exposed and unexposed groups.

Note: An Explanation and Elaboration article discusses each checklist item and gives methodological background and published examples of transparent reporting. The STROBE checklist is best used in conjunction with this article (freely available on the Web sites of PLoS Medicine at http://www.plosmedicine.org/, Annals of Internal Medicine at http://www.annals.org/, and Epidemiology at http://www.epidem.com/). Information on the STROBE Initiative is available at http://www.strobe-statement.org.

For peer review only
